# Supplementary material for: Examining the effect of non-specialised clinical rotations upon medical students’ Thanatophobia and Self-efficacy in Palliative Care: a prospective observational study in two medical schools
Source: BMJ Open. 2020 Nov 18;10(11):e041144. doi: 10.1136/bmjopen-2020-041144 (PMC7677329; doi:10.1136/bmjopen-2020-041144)
Supplement: Supplementary data [file bmjopen-2020-041144supp001.pdf]

Appendix

Survey model/ Portuguese-BR version of SEPC and Thanatophobia Scale	
Original Questions	*English Translation*
Você já perdeu na morte alguém que, segundo a sua percepção, teve um papel importante na sua vida?	**Has anybody that you have been close to died?**
Você já viu alguém morrendo?	**Have you ever witnessed a person's death?**
Durante sua formação profissional, você já participou do cuidado de alguém que acabou morrendo?	**During your professional training, have you ever taken care of anybody that eventually died?**

Medical Schools` Placements

In **Medical School 1**, students rotate in three different clinical environments. In the first 4 weeks, they stay in the Emergency Department. For an international audience, it is important to explain that Emergency Departments in Brazil are chaotic environments that are always working 4-5 times above their capacities. For instance, in medical school 1, the Emergency Department was planned to have 25 patients in observation but end up admitting more than 100. Ideally, patients should stay there for 24 hours maximum, but occasionally they stay there for up to two weeks. Even patient who need intensive care can stay in the Emergency Department for up to 5 days waiting for a vacancy inside the hospital. Students engage actively in the process of care and take direct responsibility for patients, always under the supervision of an experienced physician. In the following 4 weeks, students stay for 2 weeks in an ICU, and 2 weeks in an internal medicine with a high level of complexity, both devoted to patients admitted through the Emergency Department. In both, the internal medicine ward and ICU, in the morning, students are responsible for presenting the case of the patients during the daily clinical round and for organizing and implementing the therapeutic plan that is discussed together with the multi-professional team. On Mondays and Fridays mornings, after the round, students engage in a clinical ethics discussion about one of the patients they are caring for. In the afternoon, on Wednesdays and Thursdays, students engage in simulation activities dedicated to address communication issues and develop emotional competence. Every Friday afternoon, students have discussions about death and dying and palliative care. The structure of the weeks in the mentioned rotations are shown below.

**MS1 – Week Planning**	**Monday**	**Tuesday**	**Wednesday**	**Thursday**	**Friday**
**Weeks 1 to 4 Morning**	Emergency Department	Emergency Department	Emergency Department	Emergency Department	Emergency Department
**Afternoon**	Emergency Department	Emergency Department	Emergency Department	Emergency Department	Emergency Department
**Weeks 4 to 8 Morning**	ICU Clinical Round Ethics Discussion	ICU Clinical Round	ICU Clinical Round	ICU Clinical Round Ethics Discussion	ICU Clinical Round
**Afternoon**	Lectures	Lectures	Simulation Sessions	Simulation Sessions	Discussions about death and dying

Medical School 1 – Placement Summary

**Placement**	Emergency and Critical Care Medicine
**Length**	8 weeks
**Program hours**	440 hours
**Learning Outcomes related to Palliative Care**	- Identify terminal patients and understand the moment to shift from a predominant curative approach to a predominant palliative approach.<br>- Provide end-of-life care to terminal patients<br>- Understand the concepts of ortothanasia and dysthanasia<br>- Understand and apply the ethical principles of surrogate decision-making<br>- Understand and apply the principles of interprofessional collaboration in the context of end-of-life care<br>- Understand how the different cultural perspectives related to death and dying impact on end-of-life care<br>- Understand how our own perspectives on death and dying may interfere with the end-of-life care that we provide to our patients<br>- Develop patient-centered communication based on presence, improvisation, and empathy.
**Groups size**	14 students
**Learning activities**	- Daily rounds to: emergency department and critical care wards;<br>- Case discussion sessions;<br>- High fidelity simulation sessions: emergency room and consultation scenarios;<br>- Point-of-care ultrasound classes and bedside training;<br>- Remote learning: digital educational plataform Moodle;<br>- Mechanical ventilation classes;<br>- Palliative care classes;<br>- Palliative care ethics discussions;
**Palliative care topics**	- Breaking bad news;<br>- End-of-life ethics;<br>- Thanatology;
**Total Supervisors**	All Medical doctors<br>- Emergency department: 8<br>- Internal Medicine Ward: 2<br>- Intensive Care Unit: 2
**Supervisors experience in Medical Education**	The course coordinator is a researcher in medical education, and 2 of the eight supervisors have a Ph.D. in medical education focused on developing new active learning strategies. All the supervisors have consistently participated in medical education congresses, presenting both research and educational innovations.
**Supervisors experience in Palliative Care**	Lecturer in Palliative care topics<br>End of life care ethics discussions in weekly basis;

**Educational Methods**	- Daily wards rounds;<br>- Blended methods: classes and remote learning;<br>- Bedside teaching;<br>- Simulation sessions;<br>- Clinical case discussions;
**Learning activities related to PC and end-of-life care**	- Clinical simulation targeting breaking bad news, communication skills, and emotional competence (4 sessions of 4h each);<br>- End of Life Ethics discussions (4 sessions of 1 hour each);
**Teaching highlights**	- Simulation and feedback<br>- Reflexive learning<br>- Case discussion of complex and critical patients
**Multidisciplinary team coworking**	Coworking close to other medical specialties: psychiatry, neurology, orthopedics and traumatology
**Supervisors Feedback routine**	Daily feedback in wards rounds and other learning activities, such as Moodle and simulation sessions.<br>Grades mailing in the end of the placement with all evaluation results
**Evaluation**	Multidimensional evaluation<br>- Two theorical classic tests: multiple choice and open questions<br>- OSCE: clinical scenarios based in placement daily situations<br>- Supervisors, peers and self-evaluation on attitudes and behavior

In **Medical School 2** the Anesthesiology and Pain Medicine placement have a total of 280 hours of activities. About 140 hours of this workload is related to palliative care topics along pain medicine learning activities. The group of students are divided into sub-groups composed of 2 students to palliative care activities, which are performed during home visits, in wards and in outpatient clinics, assisting patients under palliative care. All these clinical-practical activities take place in multiprofessional settings with the presence of nurses, social workers and other professionals. At the same time there are theoretical activities in which subjects such as the treatment of acute and chronic pain, bases of palliative care and health communication are addressed, with an emphasis on bad news.

**MS2 – Week Planning**	**Monday**	**Tuesday**	**Wednesday**	**Thursday**	**Friday**
**Weeks 1 to 3 Morning**	Anesthesiology department	Anesthesiology department	Anesthesiology department	Anesthesiology department	Anesthesiology department
**Afternoon**	Anesthesiology department	Anesthesiology department	Anesthesiology department	Anesthesiology department	Anesthesiology department
**Weeks 3 to 6 Morning**	Pain and Palliative Care Outpatient Clinic	Pain and Palliative Care Outpatient Clinic	Pain and Palliative Care Outpatient Clinic	Pain and Palliative Care Outpatient Clinic	Pain and Palliative Care Outpatient Clinic
**Afternoon**	Lecture	Lecture	Clinical case discussion	Pain and Palliative Care Outpatient Clinic	Homecare

Medical School 2 – Placement Summary

**Clinical placement**	Anesthesiology and Pain Medicine
**Length**	6 weeks
**Total program hours**	280 hours
**Learning Outcomes related to Palliative Care**	- Identify patients who can benefit from palliative care<br>- Understand the concepts of palliative care<br>- Understand the importance of adequate communication in health and symptoms management<br>- Evaluate and manage acute and chronic pain
**Groups size**	12 students
**Learning activities**	- Traditional and interactive lectures using Kahoot (game-based learning platform)<br>- Patient care in outpatient clinic, in the hospital yards and home care<br>- Clinical cases discussion
**Palliative care topics**	- Palliative care concepts<br>- Communication in palliative care<br>- Pain and symptom management
**Total Supervisors**	- Medical doctors: 2<br>- Registered Nurses: 2
**Supervisors experience in Medical Education**	Medical Doctors are PhD, working as clinical teachers at the medical school for more than 10 years
**Supervisors experience in Palliative Care**	Medical Doctors are board-certificated palliative care specialists<br>Registered Nurses have no specific training
**Educational Methods**	- Lectures<br>- Clinical case discussions<br>- Training procedures skills<br>- Clinical exposure to palliative patient care
**Learning activities related to PC and end-of-life care**	- PC lectures (8h): concepts, communications skills, symptoms management<br>- Clinical discussions of outpatient patients (20h)<br>- Homecare visits (4h)
**Teaching highlights**	- Lectures<br>- Case discussions of chronic patients<br>- Working in a multi-professional healthcare team specialized in Palliative Care
**Multidisciplinary team coworking**	Discussion of clinical home care cases with the participation of registered nurse, social worker, psychologist and nutritionist. Physical therapists are available under referral
**Supervisors Feedback routine**	During daily practical activities
**Evaluation**	Classical style tests and clinical skill practical evaluation using simulated setting activities
