# Supplementary material for: Examining the effect of non-specialised clinical rotations upon medical students’ Thanatophobia and Self-efficacy in Palliative Care: a prospective observational study in two medical schools
Source: BMJ Open. 2020 Nov 18;10(11):e041144. doi: 10.1136/bmjopen-2020-041144 (PMC7677329; doi:10.1136/bmjopen-2020-041144)
Supplement: Author's manuscript [file bmjopen-2020-041144.draft_revisions.pdf]

BMJ Open

BMJ Open is committed to open peer review. As part of this commitment we make the peer review history of every article we publish publicly available.

When an article is published we post the peer reviewers' comments and the authors' responses online. We also post the versions of the paper that were used during peer review. These are the versions that the peer review comments apply to.

The versions of the paper that follow are the versions that were submitted during the peer review process. They are not the versions of record or the final published versions. They should not be cited or distributed as the published version of this manuscript.

BMJ Open is an open access journal and the full, final, typeset and author-corrected version of record of the manuscript is available on our site with no access controls, subscription charges or pay-per-view fees (http://bmjopen.bmj.com).

If you have any questions on BMJ Open's open peer review process please email info.bmjopen@bmj.com

BMJ Open

Examining the effect of specialized and non-specialized clinical rotations upon medical students' Thanatophobia and Self-efficacy in Palliative Care: a prospective observational study

Journal:	*BMJ Open*
Manuscript ID	bmjopen-2020-041144
Article Type:	Original research
Date Submitted by the Author:	31-May-2020
Complete List of Authors:	Gryschek, Guilherme; State University of Campinas Faculty of Medical Sciences, Internal Medicine<br>Cecilio-Fernandes, Dario; State University of Campinas Faculty of Medical Sciences,<br>Barros, Guilherme; Universidade Estadual Paulista Júlio de Mesquita Filho Câmpus de Botucatu Faculdade de Medicina, Department of Anesthesiology<br>Mason, Stephen; University of Liverpool Marie Curie Palliative Care Institute Liverpool<br>de Carvalho-Filho, Marco Antonio; University Medical Centre Groningen, CEDAR - Center for Educational Development and Research in Health Sciences; State University of Campinas Faculty of Medical Sciences, Internal Medicine
Keywords:	MEDICAL EDUCATION & TRAINING, PALLIATIVE CARE, Adult palliative care < PALLIATIVE CARE

SCHOLARONE™
Manuscripts

BMJ

I, the Submitting Author has the right to grant and does grant on behalf of all authors of the Work (as defined in the below author licence), an exclusive licence and/or a non-exclusive licence for contributions from authors who are: i) UK Crown employees; ii) where BMJ has agreed a CC-BY licence shall apply, and/or iii) in accordance with the terms applicable for US Federal Government officers or employees acting as part of their official duties; on a worldwide, perpetual, irrevocable, royalty-free basis to BMJ Publishing Group Ltd ("BMJ") its licensees and where the relevant Journal is co-owned by BMJ to the co-owners of the Journal, to publish the Work in this journal and any other BMJ products and to exploit all rights, as set out in our licence.

The Submitting Author accepts and understands that any supply made under these terms is made by BMJ to the Submitting Author unless you are acting as an employee on behalf of your employer or a postgraduate student of an affiliated institution which is paying any applicable article publishing charge ("APC") for Open Access articles. Where the Submitting Author wishes to make the Work available on an Open Access basis (and intends to pay the relevant APC), the terms of reuse of such Open Access shall be governed by a Creative Commons licence – details of these licences and which Creative Commons licence will apply to this Work are set out in our licence referred to above.

Other than as permitted in any relevant BMJ Author's Self Archiving Policies, I confirm this Work has not been accepted for publication elsewhere, is not being considered for publication elsewhere and does not duplicate material already published. I confirm all authors consent to publication of this Work and authorise the granting of this licence.

For peer review only

Title: Examining the effect of specialized and non-specialized clinical rotations upon medical students' Thanatophobia and Self-efficacy in Palliative Care: a prospective observational study

Running Title: Palliative care education and medical students

Word count: 2800

Authors:

- Guilherme Gryschek, MD, MSc is a Ph.D. student at School of Medical Sciences – UNICAMP, Campinas-SP, Brazil; ggryschek@gmail.com

- Dario Cecilio-Fernandes, Ph.D., is a researcher in School of Medical Sciences – UNICAMP, Campinas-SP, Brazil; dario.fernandes@gmail.com

- Guilherme Antonio Moreira de Barros, MD, MSc, Ph.D. is Associate Professor, Anaesthesiology Department, Botucatu Medical School, Sao Paulo State University - UNESP, Botucatu-SP, Brazil; guilherme.am.barros@unesp.br

- Stephen R Mason, PGCHE, Ph.D., Research Lead Palliative Care Institute Liverpool, University of Liverpool, UK; stephen.mason@liverpool.ac.uk

- Marco Antonio de Carvalho Filho (corresponding author), MD, Ph.D., Associate Professor, Internal Medicine Department, School of Medical Sciences, University of Campinas; and Senior Researcher, Center for Educational Development and Research in Health Professions (CEDAR), research group LEARN, the University of Groningen, The Netherlands; m.a.de.carvalho.filho@umcg.nl. Address: Antonius Deusinglaan 1 9713AV, Groningen, The Netherlands

For peer review only - http://bmjopen.bmj.com/site/about/guidelines.xhtml

Abstract

Introduction: Including palliative care (PC) in overloaded medical curricula is a challenge, especially where there is a lack of PC specialists. We hypothesized that non-specialized rotations could provide meaningful PC learning when there are enough clinical experiences, with adequate feedback.

Objective: observe the effects of different PC educational approaches for undergraduate medical students in two different medical schools.

Design: observational prospective study;

Setting: Medical schools in South America;

Participants: 134 sixth-year medical students of two medical schools;

Methods: This was a longitudinal study that observed the development of Self-efficacy in Palliative Care and Thanatopohobia in sixth-year medical students in two different clinical rotations: a non-specialized in medical school 1 (MS1) and a specialized in medical school 2 (MS2). We enrolled 78 students in MS1 during the Emergency and Critical Care rotation when supervisors and students care for dying patients and 56 students in MS2 during the rotation in Anesthesiology, which includes a specialized PC practice.

Primary Outcomes: Self-efficacy in Palliative Care (SEPC) and Thanatophobia (TS) scales were used to assess students at the beginning and the end of the rotations.

Results: Both groups had an increase in SEPC and a decrease in TS scores. We observed higher post-pretest differences and larger effect-sizes in MS1.

Conclusion: Non-specialized rotations that consider PC competencies as core aspects of being a doctor can be as effective as specialized rotations to develop self-efficacy in PC and decrease Thanatophobia levels.

Article Summary: Strengths and limitations of this study

For peer review only - http://bmjopen.bmj.com/site/about/guidelines.xhtml

- This study access the educational effect on students self-efficacy in palliative care of two medical schools with different curricula and educational approaches.
- Demonstrated that the experiential and reflexive learning are as important as dedicated clinical rotations in palliative care education for medical students.
- Understanding palliative care as part of the professional role and including its core competencies in diverse non-specialized clinical rotations is a possible alternative for training future doctors and healthcare professionals in palliative care.

Keywords: Medical Education, Undergraduate; Care, Palliative; Self-assessment; Self-Efficacy; Teaching & Learning;

Introduction

Medicine has made significant advances in improving healthcare outcomes during the last century. While life expectancy has increased in many countries, chronic and degenerative diseases have become the leading causes of death (1,2). To deal with this new epidemiological context, doctors need to become competent to deliver palliative and end of life care (2–4). To prepare doctors to cope with this new reality of practice, medical educators have the responsibility to develop pedagogical strategies to guide medical students towards becoming competent in supporting the psychological, social and spiritual needs of dying patients and their families.

The social cognitive theory suggests that individuals learn and change behaviour through intra-personal motivational constructs, known as 'self-efficacy' and 'outcome expectancy.' "Self-efficacy" relates to the confidence an individual (in this case, medical students) has in their capacity in performing a specific task/behaviour. Students' knowledge, skills, and previous experience, including the observation of other's performance, modulates their self-efficacy (5). Outcome expectancy refers to the

For peer review only

perceived consequence of the target behaviour, together with the aggregate value this behaviour brings to the student. A 'positive' performance is more likely when students' self-efficacy is higher, and the expected outcome is rewarding (5). Thus, appropriate training should not only increase students' knowledge, but also strengthen their self-confidence in achieving their objectives (self-efficacy), while showing the value of the desirable actions (outcome expectancies)(5,6). Consequently, based on this theory, evaluating the design of learning activities should incorporate the measurement of self-efficacy and outcome expectancy.

The Self-efficacy in Palliative Care (SEPC) and the Thanatophobia (TS) scales were developed to evaluate student's self-efficacy and outcome expectancies in palliative care (PC) (6–8). The SEPC covers core aspects of doctors' expected behaviours in PC: effective communication with the patient and family; assessment and management of patient's symptoms and needs; and multidisciplinary work within a healthcare team (7). Thanatophobia (literally 'fear of death') relates to healthcare professionals' avoidance of terminally ill patients (9). Lower levels of thanatophobia are considered desirable when healthcare professionals are providing end-of-life care; therefore, the TS scale may be used to measure the outcomes expectancies related to PC education (7,9). Both scales (SEPC and TS) were validated to measure the impact of medical training in PC learning (7).

Although medical schools include palliative care in their curricula, the teaching and assessment strategies are heterogenous, and many schools do not have access to specialized PC centers for students' placements. Examples of specialized services are hospices, wards, and outpatients' clinics run by palliative medicine specialists. In our article, we explore whether a focus on palliative care cases during non-specialized clinical rotations without the active participation of PC specialists may offer efficient learning opportunities for senior medical students and bridge a gap within existing curricula.

For peer review only - http://bmjopen.bmj.com/site/about/guidelines.xhtml

Engagement and learning within a specialized practice may be considered ideal for training purposes. However, one can argue that effective learning is mostly related to how any given scenario is modulated by students and supervisors to optimize the learning process the learning process through different lenses. Among these lenses, the Kolb's cycle offers a compelling insight into understanding the learning process triggered by practical experiences. Kolb's cycle stresses the importance of integrating the practical experience with the abstract conceptualization of this experience. Therefore, the learning process occurs through consecutive cycles of lived experiences, reflexive observations, and active planning of the next experimentations supported by appropriate supervision and feedback (10). Thus, we hypothesize that non-specialized centers can offer meaningful and productive clinical experiences capable of grounding the improvement of self-efficacy in PC and the decreasing of thanatophobia levels.

To verify our hypothesis, we assessed PC training outcomes in two different medical schools in Brazil. The first medical school offers a clinical rotation without a specialized service, but with opportunities to reflect and discuss the care for dying patients. The clinical supervisors within this first school (MS1) are not specialized in PC care, but they accept end-of-life care as part of their professional and educational role as general internal medicine doctors. The second medical school (MS2) offers a clinical rotation in a PC specialized service focused on ambulatory care and a homecare setting, both relying on specialized PC supervision. In this longitudinal study, we aimed to measure medical students' self-efficacy in PC and thanatophobia levels, before and after those two different rotations in the aforementioned medical schools.

Methods

Context

The undergraduate medical course in Brazil is six years long. National guidelines for curriculum development demand the integration of learning activities with healthcare

For peer review only - http://bmjopen.bmj.com/site/about/guidelines.xhtml

services throughout the training. In general, during the first two years, students attend classroom-based teaching, with a combination of lectures and small group discussions. Besides the classroom activities, students have regular placements in primary care and hospital facilities to be introduced to the logic of patient care. During years three and four, students are trained in history taking, physical examination and clinical reasoning, and they are involved in the longitudinal care of patients in primary care facilities. During the last two years, students undertake an internship, where they rotate in diverse clinical placements such as internal medicine, paediatrics, primary care, gynaecology, surgery, critical care, and medical emergencies. In these clinical rotations, students work under supervision and take direct responsibility for patient care in clinical and surgical wards, outpatient clinics, and emergency departments. After concluding their undergraduate studies, they receive full registration to practice medicine and are allowed to perform independently, even without resident training. Many new doctors start working at emergency departments and primary care facilities, despite their lack of postgraduate training.

Design, Setting and Participants

We conducted a longitudinal observational study with two cohorts of Year 6 medical students, from two separate Medical Schools, in 2017. Both medical schools are ranked in the top 10 within Brazil (11,12).

Medical School 1 (MS1): final year students undertake an 8-week mandatory emergency and critical care placement. During this rotation, students engage in clinical activities for 11 hours daily, under the direct supervision of clinical teachers, and experience the care of chronically ill and dying patients. The clinical supervisors decided to include PC discussions in bedside rounds due to the opportunities provided by this placement. The supervisors also provide formally structured discussions about end-of-life ethics. Also, students have access to training sessions with simulated patients to develop communication skills (including breaking bad news) and emotional competence

For peer review only - http://bmjopen.bmj.com/site/about/guidelines.xhtml

(13,14). As such, students have several opportunities to acquire the knowledge, skills, and attitudes required to care for dying patients, and reflexive learning is stimulated throughout the rotation by the supervisors. Nevertheless, most of the discussions during the rotation are about emergency medicine and critical care, and the activities around the topic of PC are complementary.

Medical School 2 (MS2): final year medical students undertake a 3-week mandatory rotation in anaesthesiology and pain therapy. Within this period, students have 32 hours dedicated to PC. Learning opportunities include lectures on PC concepts and pain management, home care visits for PC patients and supervised activities in the PC outpatient clinic under the supervision of PC consultants. In this placement, students work together with the PC multi-professional team, which includes social workers, psychologists, nurses, physical therapists, and nutritionists. The students learn how to manage symptoms, work in a multidisciplinary team, and deliver care for PC patients in different settings, such as outpatient clinics and home care. Aside from PC teaching, students also engage in learning activities on anaesthesiology.

Table 1 summarizes the organization of the rotations in MS1 and MS2.

Table 1 – Placements' Activities in MS1 and MS2

	**Medical School 1**	**Medical School 2**
**Clinical placement**	Emergency and Critical Care Medicine	Anesthesiology and Pain Medicine
**Length**	8 weeks	3.5 weeks
**Total program hours**	440 hours	140 hours
**Palliative medicine exclusive teaching**	None; PC discussions are included in regular clinical activities	32 hours dedicated to PC

For peer review only - http://bmjopen.bmj.com/site/about/guidelines.xhtml

**Learning activities related to PC and end-of-life care**	Clinical simulation targeting breaking bad news, communication skills, and emotional competence (4 sessions of 4h each);<br>Bedside teaching (daily rounds);<br>End of Life Ethics discussions (4 sessions of 1 hour each);	PC lectures (8h): concepts, communications skills, symptoms management;<br>Clinical discussions of ambulatory patients (20h);<br>Homecare visits (4h);
**Teaching highlights**	Simulation and feedback;<br>Reflexive learning;<br>Case discussion of complex and critical patients;	Lectures;<br>Case discussions of chronic patients;<br>Working in a multi-professional healthcare team specialized in Palliative Care;

Procedure

Data were collected from February 2017 to September 2017, when we send the invitation for participation in the study to all the students enrolled in the aforementioned rotations at MS1 and MS2 (80 and 60 students respectively). The main author (GG) had no participation in the rotations and personally invited the students during the introductory activities of each rotation. GG explained to students the research goals, the nature of the instruments that would be applied and guaranteed the anonymity of the participants. Students who agreed to participate signed a written informed consent. We collected data from each group of students (MS1 and MS2) in two different time-points of their placements: the first point was at the start of the placement; the second one was during the last week.

Questionnaires

For peer review only - http://bmjopen.bmj.com/site/about/guidelines.xhtml

Demographic survey: The authors developed a demographic questionnaire to explore students' previous personal and professional experiences related to palliative and end-of-life care (Appendices), as it was expected that past experiences would influence students' learning and performance (15,16).

Self-efficacy in Palliative Care Scale (SEPC) (7): The SEPC measures the self-perceived efficacy in PC by recording students' ratings of their confidence in performing PC related actions, using a 100 mm Visual Analogue Scale. The visual analogue scales range from 'would feel very anxious' to 'would feel very confident.' The point assigned on the visual analog scale is measured and scored from zero up to 100. The 23-item SEPC has three sub-scales of a) Communication (8 items; score ranging from 0 to 800); b) Patient management (8 items; score ranging from 0 to 800); c) Multi-disciplinary team working (7 items; score ranging from 0 to 700). The original scale was translated and validated to Brazilian Portuguese previously to this study (in press).

Thanatophobia Scale (9): The original TS has seven items and assesses attitudes related to the care for dying patients. The scale offers a reliable measure of students' outcome expectancy related to PC (7). Participants rate how much they agree/disagree with expressed negative attitudinal statements, with the final scores ranging from 7 to 49. Higher ratings indicate higher thanatophobia levels. The original scale was translated and validated to Brazilian Portuguese before this study (in press).

Ethics

This research was conducted following the Declaration of Helsinki. We assured any student who was uncomfortable with the research enquiring would not feel obliged to participate. Themes related to death could be emotive and/or sensitive; thus, students could contact the researchers to receive support or referral if/as required. The Research Ethics Committee of both medical schools approved the study (58198016.4.0000.5404/2016; 58198016.4.3001.5411/2016).

For peer review only - http://bmjopen.bmj.com/site/about/guidelines.xhtml

Data Analysis

Continuous variables were described by mean, minimal and maximal values, and categorical variables as absolute (n) and relative frequency (%). We analyzed the difference between students' pre and post main scores on the scales using t-tests. Cohen's d and effect size were used for checking the amplitude of changes in pre- and post-test scores, providing an index to assess the magnitude of the difference detected on t-test results. The effect size was characterized as small, medium, large, or very large and evaluated the magnitude of change despite the sample size. We also compared between MS1 and MS2 students' pre and post scores using t-tests. Data were analyzed using IBM-SPSS 21.0 (17).

Results

From the total students invited, two students from each school were excluded due to incomplete data. Thus, we included 78 students from MS1 and 56 students in MS2 (65.0% and 62.2% of last year students, respectively). Students' demographic data and previous experiences are summarized in Table 2.

Table 2 - Descriptive data of MS1 and MS2

	Medical School 1	Medical School 2
**Sample size**	78	56
**Average age in years (min – max)**	24.8 (22-30)	25.2 (22-34)
**Gender**		
Male	36 (46.2%)	30 (53.6%)
Female	42 (53.8%)	26 (46.4%)
Has anybody that you have been close to died?		
Yes	63 (80.8%)	45 (80.4%)

For peer review only - http://bmjopen.bmj.com/site/about/guidelines.xhtml

No	15 (19.2%)	11 (19.6%)
Have you ever witnessed the moment of a person's death?		
Yes	64 (82.1%)	52 (92.9%)
No	14 (17.9%)	4 (7.1%)
During your professional training, have you ever taken care of anybody that eventually died?		
Yes	75 (96.2%)	55 (98.2%)
No	3 (3.8%)	1 (1.8%)

In both schools, students had a significant increase in self-efficacy in all dimensions of the SEPC ($p<0.001$ for all analyses). Students' thanatophobia levels significantly decreased in MS1 ($p<0.001$), but not in MS2 ($p<0.05$) – see table 3. The effect size of the change in SEPC was larger at MS1 when compared to MS2, except on the SEPC Patient Management subscale, which nevertheless showed a very large effect size in both schools. The effect size magnitude of TS was moderate in MS1.

Table 3 – SEPC and Thanatophobia score means, t-test and effect size at Medical School 1 and 2

Instruments	***Pre-test***	***Post-test***	***Difference Post - Pre***	***t***	***p***	***CI95%***	***Cohen's d***	***Effect Size Magnitude***
**Medical School 1 (n=78)**								
SEPC - Communication	346.50 (145.17)	573.90 (124.47)	227.39 (138.50)	14.50	**<0.001**	**258.62, 196.17**	**1.64**	***Very large***
SEPC - Patient Management	338.38 (112.56)	527.50 (113.55)	189.11 (140.30)	11.90	**<0.001**	**220.75, 157.48**	***1.35***	***Very large***

For peer review only - http://bmjopen.bmj.com/site/about/guidelines.xhtml

SEPC - Multidisciplinary Teamwork	361.26 (124.28)	470.35 (105.77)	109.09 (118.86)	8.11	**<0.001**	**135.17, 81.01**	***0.92***	***Large***
TS	21.54 (7.56)	16.54 (7.58)	-5.00 (5.91)	-7.06	**<0.001**	**3.59, 6.41**	***0.79***	***Moderate***
**Medical School 2 (n=56)**								
**SEPC - Communication**	296.41 (147.41)	398.00 (171.64)	101.59 (151.30)	5.02	**<0.001**	**142.11, 61.07**	**0.67**	***Moderate***
**SEPC - Patient Management**	327.86 (133.91)	462.07 (126.55)	134.21 (132.69)	7.57	**<0.001**	**169.75, 98.68**	**1.01**	***Very Large***
**SEPC - Multidisciplinary Teamwork**	388.50 (120.18)	454.96 (120.49)	66.46 (136.82)	3.63	**=0.001**	**103.10, 29.83**	**0.49**	***Small***
**TS**	22.82 (8.77)	21.20 (7.67)	1.62 (5.45)	1.96	**<0.05**	**-0.14, -3.07**	**0.26**	***Small***

We did not find significant differences among the pre-test scores from both scales when comparing MS1 with MS2. At the post-test, however, we find differences between SEPC Communication and Patient Management subscales (CM-MS1 post = 573.90 x CM-MS2 post = 398.00, p<0.001 and PM-MS1 post = 527.50 x PM-MS2 post = 462.55, p=0.002) and in TS (MS1 post=16.54 x MS2 post=21.20, p<0.001) (Table 4).

Table 4 – Medical schools' comparisons: score means and t-test results

		MS1	MS2	t	p	CI95%
SEPC – **Communication (CM)**	**Pre**	346.50 (145.17)	296.41 (147.41)	1.96	0.052	-0.53, 100.71
	**Post**	573.90 (124.47)	398.00 (171.64)	**6.53**	**<0.001**	**122.45, 229.34**

For peer review only - http://bmjopen.bmj.com/site/about/guidelines.xhtml

SEPC – **Patient Management** (PM)	**Pre**	338.38 (112.56)	327.86 (133.91)	0.49	0.62	-31.71, 52.76
	**Post**	527.50 (113.75)	462.07 (126.55)	**3.13**	**0.002**	**24.11, 106.75**
SEPC – **Multidisciplinary teamwork (MT)**	**Pre**	361.26 (124.28)	388.50 (120.18)	-1.27	0.21	-69.71, 15.23
	**Post**	470.35 (105.77)	454.96 (120.49)	0.78	0.43	-23.47, 54.15
TS	**Pre**	21.49 (7.53)	22.72 (8.46)	-0.90	0.37	-3.97, 1.50
	**Post**	16.54 (7.58)	21.20 (7.67)	**-3.50**	**<0.001**	**-7.28, -2.02**

Discussion

As a consequence of an aging population and a higher prevalence of chronic illness that impacts on quality and life expectancy, PC has become an essential clinical competency for all doctors. Ultimately, clinicians in most specialties, such as internal medicine, geriatrics, oncology, emergency medicine, intensive care, primary care, paediatrics, and so on, will have PC patients among their caseloads. Including PC in the already busy and crowded medical curriculum poses a challenge for medical educators. The challenge has two edges; one is the scarcity of specialized services and professionals to facilitate training, particularly in the developing world (where the PC is still incipient and needs substantial development); the second is the lack of space in the curriculum.

Worldwide, medical educators have devised different strategies for PC training, and there is no 'gold standard' to guide best practices (18–20). Although there can often be a lack of PC services, different disciplines and placements should include PC in their curricula and provide PC training for all medical students (21). Our study showed that

For peer review only

For peer review only - http://bmjopen.bmj.com/site/about/guidelines.xhtml

both clinical rotations, with and without specific placements in PC, were capable of improving students` self-efficacy in PC and decreasing their thanatophobia levels.

Interestingly, although MS1 did not have specific activities devoted to palliative care, the effect size and the final score improvements in both scales were significantly higher for this group of students. We believe that this finding is related to opportunities to reflect on real end-of-life cases that were provided for the students during this rotation. As anticipated by Kolb`s experiential learning theory, the several occasions to reflect in and on the practice nurtured confidence and a readiness to practice PC. Moreover, clinical supervisors at MS1 embraced caring for dying patients as a core aspect of their practice despite their lack of specialized training as palliative medicine doctors. This positive role-modelling, allied to the experiential and reflexive learning, may explain the increase in students' self-efficacy in PC observed at the end of the rotation. In fact, the social cognitive theory states that self-efficacy and outcome expectancy are both modulated by the perceived support from supervisors, which will eventually, impact on the internalization of the desired behaviour (6). We conclude that offering opportunities to experience and reflect, guided by qualified supervision with structured feedback, may be more important than having access to specialized PC centres for the learning of undergraduate students.

The challenge posed to medical educators is building up a meaningful learning process in complex workplaces in different clinical rotations, despite the lack of ideal circumstances (10). Clinical teachers need to nurture the learning experience of students embracing the contextual elements, even the negative ones, as teaching assets and not as pitfalls. Students will practice medicine in the real world and need to learn how to deal with the context of the workplace. Those with higher self-efficacy are more likely to reflect on their challenges and experiences to improve performance (5). In this sense, reflection is essential not only to learn but also to feel the urge to change and improve the healthcare delivered to patients (22). Therefore, including structured PC discussions in clinical rotations may offer an opportunity for teachers not only to prepare students to

For peer review only - http://bmjopen.bmj.com/site/about/guidelines.xhtml

provide proper palliative care but also to open the debate about the relevance of including PC into their future practice.

Strengths and limitations

This study had a consistent methodological approach and involved two different medical schools, with different curricula to delivery PC education to medical undergraduate students. We demonstrated that, although the two groups of students had different approaches in the sense of learning PC, both groups had improvements in their self-efficacy, even when did not have access to specialized training in PC.

As with all research, this study has some limitations. We did not access students' conceptual knowledge of PC. Although the students have expressed more confidence in their performance, we cannot determine how and if their performance improved. Also, we cannot conclude that the improvements in SEPC and thanatophobia, observed in this study, will be persistent for the actual medical practice of the students. The proper assessment for this will require further evaluation over the new doctors in their practice.

Implications for practice and next steps

In a context of an overloaded curriculum and a lack of departments and services of palliative medicine in medical schools, a vertical insertion of PC concepts and practices along the course, in different disciplines and rotations, could address PC learning needs for undergraduate students, even without specialized PC doctors and services.

We still need to understand the impact of implementing PC training on clinical rotations on students` declarative and procedural knowledge acquisition. It is also essential to clarify what are the teaching methodologies related to better development of PC competencies in undergraduate medical students.

Conclusion

For peer review only - http://bmjopen.bmj.com/site/about/guidelines.xhtml

Our findings support that non-specialized rotations can include PC basic competences as part of the training. Medical schools could prepare future doctors to provide PC in any area it is required by embracing palliative care as a core competence, indispensable for becoming a doctor.

Glossary Terms

Palliative care: "approach that improves the quality of life of patients and their families facing the problem associated with life-threating illness, through the prevention and relief of suffering by means of early identification and impeccable assessment and treatment of pain and other problems, physical, psychosocial and spiritual."(2)

Self-efficacy: "the confidence to carry out the courses of action necessary to accomplish desired goals" (23)**.**

Thanatophobia: death anxiety that caregivers and healthcare professionals may feel when caring for dying patients (9).

Declarations

Ethics approval and consent to participate

This research was conducted following the Declaration of Helsinki. We assured any student who was uncomfortable with the research enquiring would not feel obliged to participate. Themes related to death could be emotive and/or sensitive; thus, students could contact the researchers to receive support or referral if/as required. The Research Ethics Committee of both medical schools approved the study (58198016.4.0000.5404/2016; 58198016.4.3001.5411/2016).

Consent for publication

Not applicable

Availability of data and materials

For peer review only - http://bmjopen.bmj.com/site/about/guidelines.xhtml

The datasets used and analysed during the current study are available from the corresponding author on reasonable request.

Competing interests

The authors declare that they have no competing interests.

Funding

This work was supported by CAPES, grant number 88881.188776/2018-01 (PhD sandwich scholarship – Edital 47/2017) to the first author for analysis and manuscript writing.

Author contributions

GG, SRM and MACF conceived and designed the study. GG and GAMB collected the data. GG, DCF and MACF analysed the data. GG and MACF were the major contributors in manuscript writing. DCF, GAMB and SRM provided meaningful inputs and critical review of the manuscript. All authors read and approved the final manuscript.

Acknowledgements

Palliative Care Institute Liverpool, the University of Liverpool for hosting and supporting the manuscript writing;

Medical students and supervisors involved on data collection;

Author's information

GG, M.Sc., M.D., G.P. is a PhD student at the School of Medical Sciences, UNICAMP, Brazil, in the field of medical education focused on palliative care education;

DCF, PhD is a young investigator at Department of Medical Psychology and Psychiatry, School of Medical Sciences, UNICAMP, Brazil;

For peer review only - http://bmjopen.bmj.com/site/about/guidelines.xhtml

For peer review only

GAMB, MD, MSc, Ph.D. is Associate Professor, Anaesthesiology Department, Botucatu Medical School, Sao Paulo State University - UNESP, Botucatu-SP, Brazil

SRM, PhD is Research Lead at Palliative Care Institute Liverpool, University of Liverpool, Liverpool, UK;

MACF, M.D., PhD is Associate Professor of Emergency Medicine at the School of Medical Sciences, the University of Campinas, Brazil and Senior Researcher at Center for Education Development and Research in Health Professions (CEDAR), University of Groningen, Groningen, the Netherlands;

References

1. Marinho F, de Azeredo Passos VM, Carvalho Malta D, Barboza França E, Abreu DMX, Araújo VEM, et al. Burden of disease in Brazil, 1990–2016: a systematic subnational analysis for the Global Burden of Disease Study 2016. Lancet [Internet]. 2018 [cited 2018 Dec 14];392(10149):760–75. Available from: https://vizhub.

2. Connor SR, Sepulveda Bermedo MC. Global atlas of palliative care at the end of life [Internet]. World Palliative Care Alliance. 2014. 111 p. Available from: http://www.who.int/nmh/Global_Atlas_of_Palliative_Care.pdf

3. Murtagh FE, Bausewein C, Verne J, Groeneveld EI, Kaloki YE, Higginson IJ. How many people need palliative care? A study developing and comparing methods for population-based estimates. Palliat Med [Internet]. 2014 [cited 2019 Mar 6];28(1):49–58. Available from: https://journals.sagepub.com/doi/pdf/10.1177/0269216313489367

4. Etkind SN, Bone AE, Gomes B, Lovell N, Evans CJ, Higginson IJ, et al. How many people will need palliative care in 2040? Past trends, future projections and implications for services. BMC Med [Internet]. 2017 [cited 2019 Mar 6];15:102. Available from: https://www.ncbi.nlm.nih.gov/pmc/articles/PMC5436458/pdf/12916_2017_Article

For peer review only - http://bmjopen.bmj.com/site/about/guidelines.xhtml

_860.pdf

5. Artino AR. Academic self-efficacy: from educational theory to instructional practice. Perspect Med Educ [Internet]. 2012 [cited 2019 Feb 20];1(2):76–85. Available from: https://link.springer.com/content/pdf/10.1007%2Fs40037-012-0012-5.pdf

6. Mason SR, Ellershaw J. Preparing for palliative medicine; evaluation of an education programme for fourth year medical undergraduates. Palliat Med [Internet]. 2008 [cited 2019 Feb 11];22:687–92. Available from: https://journals.sagepub.com/doi/pdf/10.1177/0269216308090070

7. Mason S, Ellershaw J. Assessing undergraduate palliative care education: Validity and reliability of two scales examining perceived efficacy and outcome expectancies in palliative care. Med Educ. 2004;38(10):1103–10.

8. Neil H, Frey R, Gott M. Instruments used to measure the effectiveness of palliative care education initiatives at the undergraduate level: A systematic literature review. BMJ Support Palliat Care [Internet]. 2013 [cited 2019 Feb 11];3:114–9. Available from: http://www.sherpa.ac.uk/romeo/issn/2045-435X/https://researchspace.auckland.ac.nz/docs/uoa-docs/rights.htm

9. Merrill J, Lorimor R, Thornby J, Woods A. Caring for Terminally ILL Persons: Comparative Analysis of Attitudes (Thanatophobia) of Practicing Physicians, Student Nurses, and Medical Students. Psychol Rep. 1998;

10. Yardley S, Teunissen PW, Dornan T. Experiential learning: AMEE Guide No. 63. Med Teach. 2012 Feb;34(2).

11. Medicina - Ranking de Cursos - Ranking Universitário Folha - 2018 [Internet]. [cited 2019 May 9]. Available from: https://ruf.folha.uol.com.br/2018/ranking-de-cursos/medicina/

12. Universities | Top Universities [Internet]. [cited 2019 May 9]. Available from: https://www.topuniversities.com/universities/country/brazil/subject/medicine

13. Schweller M, Ribeiro DL, Passeri SR, Wanderley JS, Carbalho-Filho MA.

For peer review only - http://bmjopen.bmj.com/site/about/guidelines.xhtml

Simulated medical consultations with standardized patients: In-depth debriefing based on dealing with emotions. Rev Bras Educ Med [Internet]. 2018 [cited 2019 Jun 13];42(1):82–91. Available from: http://dx.doi.org/10.1590/1981-52712018v42n1RB20160089

14. Schweller M, Costa FO, Antonio MAR, Amaral EM, Carvalho-Filho MA de. The impact of simulated medical consultations in the empathy levels of students at one medical school. Acad Med [Internet]. 2014 [cited 2019 Mar 5];89(4):632–7. Available from: https://www.ncbi.nlm.nih.gov/pmc/articles/PMC4885552/pdf/acm-89-632.pdf

15. Whyte R, Quince T, Benson J, Wood D, Barclay S. Medical students' experience of personal loss: incidence and implications. BMC Med Educ [Internet]. 2013 [cited 2019 Mar 4];13:36. Available from: http://www.biomedcentral.com/1472-6920/13/36

16. Barclay S, Whyte R, Thiemann P, Benson J, Wood DF, Parker RA, et al. An important but stressful part of their future work: Medical students' attitudes to palliative care throughout their course. J Pain Symptom Manage [Internet]. 2015;49(2):231–42. Available from: http://dx.doi.org/10.1016/j.jpainsymman.2014.06.004

17. IBM Corp. IBM SPSS Statistics for Windows. Armonk, NY: IBM Corp.; 2012.

18. Fitzpatrick D, Heah R, Patten S, Ward H. Palliative Care in Undergraduate Medical Education-How Far Have We Come? Am J Hosp Palliat Med [Internet]. 2017 [cited 2019 Mar 22];34(8):762–73. Available from: https://journals.sagepub.com/doi/pdf/10.1177/1049909116659737

19. Field D, Wee B. Preparation for palliative care: Teaching about death, dying and bereavement in UK medical schools 2000-2001. Med Educ. 2002;36(6):561–7.

20. Schulz-Quach C, Wenzel-Meyburg U, Fetz K. Can e-learning be used to teach palliative care?-medical students' acceptance, knowledge, and self-estimation of competence in palliative care after elearning. BMC Med Educ [Internet]. 2018

For peer review only - http://bmjopen.bmj.com/site/about/guidelines.xhtml

[cited 2019 Mar 22];18(82). Available from: https://doi.org/10.1186/s12909-018-1186-2

21. Lloyd-Williams M, Macleod RDM, Macleod RD. A systematic review of teaching and learning in palliative care within the medical undergraduate curriculum A systematic review of teaching and learning in palliative care within the medical undergraduate curriculum. Med Teach [Internet]. 2004 [cited 2019 Feb 12];26(8):683–90. Available from: https://doi.org/10.1080/01421590400019575

22. Mason SR, Ellershaw J. Undergraduate training in palliative medicine: is more necessarily better? Palliat Med [Internet]. 2010 [cited 2019 Jan 29];24(3):306–9. Available from: https://journals.sagepub.com/doi/pdf/10.1177/0269216309351867

23. Klassen RM, Klassen JRL. Self-efficacy beliefs of medical students: a critical review. Perspect Med Educ [Internet]. 2018 [cited 2019 Feb 20];7(2):76–82. Available from: https://doi.org/10.1007/s40037-018-0411-3

For peer review only

Appendices

Survey model/ Portuguese-BR version of SEPC and Thanatophobia Scale	
Original Questions	*English Translation*
Você já perdeu na morte alguém que, segundo a sua percepção, teve um papel importante na sua vida?	**Has anybody that you have been close to died?**
Você já viu alguém morrendo?	**Have you ever witnessed a person's death?**
Durante sua formação profissional, você já participou do cuidado de alguém que acabou morrendo?	**During your professional training, have you ever taken care of anybody that eventually died?**

For peer review only

Reporting checklist for cohort study.

Based on the STROBE cohort guidelines.

Instructions to authors

Complete this checklist by entering the page numbers from your manuscript where readers will find each of the items listed below.

Your article may not currently address all the items on the checklist. Please modify your text to include the missing information. If you are certain that an item does not apply, please write "n/a" and provide a short explanation.

Upload your completed checklist as an extra file when you submit to a journal.

In your methods section, say that you used the STROBE cohortreporting guidelines, and cite them as:

von Elm E, Altman DG, Egger M, Pocock SJ, Gotzsche PC, Vandenbroucke JP. The Strengthening the Reporting of Observational Studies in Epidemiology (STROBE) Statement: guidelines for reporting observational studies.

For peer review only

		Reporting Item	Page Number
**Title and abstract**			
Title	#1a	Indicate the study's design with a commonly used term in the title or the abstract	1
Abstract	#1b	Provide in the abstract an informative and balanced summary of what was done and what was found	2
**Introduction**			
Background / rationale	#2	Explain the scientific background and rationale for the investigation being reported	3
Objectives	#3	State specific objectives, including any prespecified hypotheses	5
**Methods**			
Study design	#4	Present key elements of study design early in the paper	6
Setting	#5	Describe the setting, locations, and relevant dates, including periods of	7, 8

		recruitment, exposure, follow-up, and data collection	
Eligibility criteria	#6a	Give the eligibility criteria, and the sources and methods of selection of participants. Describe methods of follow-up.	6-8
Eligibility criteria	#6b	For matched studies, give matching criteria and number of exposed and unexposed	n/a
Variables	#7	Clearly define all outcomes, exposures, predictors, potential confounders, and effect modifiers. Give diagnostic criteria, if applicable	8
Data sources / measurement	#8	For each variable of interest give sources of data and details of methods of assessment (measurement). Describe comparability of assessment methods if there is more than one group. Give information separately for for exposed and unexposed groups if applicable.	9
Bias	#9	Describe any efforts to address potential sources of bias	8, 10
Study size	#10	Explain how the study size was arrived at	8
Quantitative variables	#11	Explain how quantitative variables were handled in the analyses. If applicable, describe which groupings were chosen, and why	10
Statistical methods	#12a	Describe all statistical methods, including those used to control for confounding	10
Statistical methods	#12b	Describe any methods used to examine subgroups and interactions	10
Statistical methods	#12c	Explain how missing data were addressed	10
Statistical methods	#12d	If applicable, explain how loss to follow-up was addressed	10
Statistical methods	#12e	Describe any sensitivity analyses	n/a
**Results**			
Participants	#13a	Report numbers of individuals at each stage of study—eg numbers potentially eligible, examined for eligibility, confirmed eligible, included in the study, completing follow-up, and analysed. Give information separately for for exposed and unexposed groups if applicable.	10

For peer review only

Participants	#13b	Give reasons for non-participation at each stage	10
Participants	#13c	Consider use of a flow diagram	n/a
Descriptive data	#14a	Give characteristics of study participants (eg demographic, clinical, social) and information on exposures and potential confounders. Give information separately for exposed and unexposed groups if applicable.	10, 11
Descriptive data	#14b	Indicate number of participants with missing data for each variable of interest	10
Descriptive data	#14c	Summarise follow-up time (eg, average and total amount)	n/a
Outcome data	#15	Report numbers of outcome events or summary measures over time. Give information separately for exposed and unexposed groups if applicable.	11-13
Main results	#16a	Give unadjusted estimates and, if applicable, confounder-adjusted estimates and their precision (eg, 95% confidence interval). Make clear which confounders were adjusted for and why they were included	11-13
Main results	#16b	Report category boundaries when continuous variables were categorized	11-13
Main results	#16c	If relevant, consider translating estimates of relative risk into absolute risk for a meaningful time period	n/a
Other analyses	#17	Report other analyses done—e.g., analyses of subgroups and interactions, and sensitivity analyses	n/a
**Discussion**			
Key results	#18	Summarise key results with reference to study objectives	13
Limitations	#19	Discuss limitations of the study, taking into account sources of potential bias or imprecision. Discuss both direction and magnitude of any potential bias.	15
Interpretation	#20	Give a cautious overall interpretation considering objectives, limitations, multiplicity of analyses, results from similar studies, and other relevant evidence.	14
Generalisability	#21	Discuss the generalisability (external validity) of the study results	15
**Other Information**			

For peer review only

Funding	#22	Give the source of funding and the role of the funders for the present study and, if applicable, for the original study on which the present article is based	17

The STROBE checklist is distributed under the terms of the Creative Commons Attribution License CC-BY. This checklist was completed on 31. May 2020 using https://www.goodreports.org/, a tool made by the EQUATOR Network in collaboration with Penelope.ai

For peer review only

BMJ Open

Examining the effect of non-specialized clinical rotations upon medical students' Thanatophobia and Self-efficacy in Palliative Care: a prospective observational study in two medical schools

Journal:	*BMJ Open*
Manuscript ID	bmjopen-2020-041144.R1
Article Type:	Original research
Date Submitted by the Author:	05-Aug-2020
Complete List of Authors:	Gryschek, Guilherme; State University of Campinas Faculty of Medical Sciences, Internal Medicine<br>Cecilio-Fernandes, Dario; State University of Campinas Faculty of Medical Sciences,<br>Barros, Guilherme; Universidade Estadual Paulista Júlio de Mesquita Filho Câmpus de Botucatu Faculdade de Medicina, Department of Anesthesiology<br>Mason, Stephen; University of Liverpool Marie Curie Palliative Care Institute Liverpool<br>de Carvalho-Filho, Marco Antonio; University Medical Centre Groningen, CEDAR - Center for Educational Development and Research in Health Sciences; State University of Campinas Faculty of Medical Sciences, Internal Medicine
**Primary Subject Heading**:	Medical education and training
Secondary Subject Heading:	Palliative care, Medical education and training
Keywords:	MEDICAL EDUCATION & TRAINING, PALLIATIVE CARE, Adult palliative care < PALLIATIVE CARE

SCHOLARONE™
Manuscripts

I, the Submitting Author has the right to grant and does grant on behalf of all authors of the Work (as defined in the below author licence), an exclusive licence and/or a non-exclusive licence for contributions from authors who are: i) UK Crown employees; ii) where BMJ has agreed a CC-BY licence shall apply, and/or iii) in accordance with the terms applicable for US Federal Government officers or employees acting as part of their official duties; on a worldwide, perpetual, irrevocable, royalty-free basis to BMJ Publishing Group Ltd ("BMJ") its licensees and where the relevant Journal is co-owned by BMJ to the co-owners of the Journal, to publish the Work in this journal and any other BMJ products and to exploit all rights, as set out in our licence.

The Submitting Author accepts and understands that any supply made under these terms is made by BMJ to the Submitting Author unless you are acting as an employee on behalf of your employer or a postgraduate student of an affiliated institution which is paying any applicable article publishing charge ("APC") for Open Access articles. Where the Submitting Author wishes to make the Work available on an Open Access basis (and intends to pay the relevant APC), the terms of reuse of such Open Access shall be governed by a Creative Commons licence – details of these licences and which Creative Commons licence will apply to this Work are set out in our licence referred to above.

Other than as permitted in any relevant BMJ Author's Self Archiving Policies, I confirm this Work has not been accepted for publication elsewhere, is not being considered for publication elsewhere and does not duplicate material already published. I confirm all authors consent to publication of this Work and authorise the granting of this licence.

For peer review only

Title: ***"Examining the effect of non-specialized clinical rotations upon medical students' Thanatophobia and Self-efficacy in Palliative Care: a prospective observational study in two medical schools"***

Running Title: Palliative care education and medical students

Word count: 3925

Authors:

- Guilherme Gryschek, MD, MSc is a Ph.D. student at School of Medical Sciences – University of Campinas (UNICAMP), Campinas-SP, Brazil; ggryschek@gmail.com

- Dario Cecilio-Fernandes, Ph.D., is a researcher the Department of Medical Psychology and Psychiatry, School of Medical Sciences – University of Campinas (UNICAMP), Campinas-SP, Brazil; dario.fernandes@gmail.com

- Guilherme Antonio Moreira de Barros, MD, MSc, Ph.D. is Associate Professor, Anaesthesiology Department, Botucatu Medical School, Sao Paulo State University - UNESP, Botucatu-SP, Brazil; guilherme.am.barros@unesp.br

- Stephen R Mason, PGCHE, Ph.D., Research Lead Palliative Care Institute Liverpool, University of Liverpool, UK; stephen.mason@liverpool.ac.uk

- Marco Antonio de Carvalho Filho (corresponding author), MD, Ph.D., Associate Professor, Internal Medicine Department, School of Medical Sciences, University of Campinas; and Senior Researcher, Center for Educational Development and Research in Health Professions (CEDAR), research group LEARN, the University of Groningen, The Netherlands; m.a.de.carvalho.filho@umcg.nl. Address: Antonius Deusinglaan 1 9713AV, Groningen, The Netherlands

For peer review only - http://bmjopen.bmj.com/site/about/guidelines.xhtml

Abstract

Introduction: Including palliative care (PC) in overloaded medical curricula is a challenge, especially where there is a lack of PC specialists. We hypothesized that non-specialized rotations could provide meaningful PC learning when there are enough clinical experiences, with adequate feedback.

Objective: observe the effects of including PC topics in non-specialized placements for undergraduate medical students in two different medical schools.

Design: observational prospective study;

Setting: Medical schools in South America;

Participants: 134 sixth-year medical students of two medical schools;

Methods: This was a longitudinal study that observed the development of Self-efficacy in Palliative Care and Thanatophobia in sixth-year medical students in different non-specialized clinical rotations in two Brazilian medical schools (MS1 and MS2). We enrolled 78 students in MS1 during the Emergency and Critical Care rotation and 56 students in MS2 during the rotation in Anaesthesiology. Both schools provide PC discussions with different learning environment and approaches.

Primary Outcomes: Self-efficacy in Palliative Care (SEPC) and Thanatophobia (TS) scales were used to assess students at the beginning and the end of the rotations.

Results: In both schools students had an increase in SEPC and a decrease in TS scores.

Conclusion: Non-specialized rotations that consider PC competencies as core aspects of being a doctor can be effective to develop self-efficacy in PC and decrease Thanatophobia levels.

Article Summary: Strengths and limitations of this study

- This study access students' self-efficacy in palliative care of two Brazilian medical schools, where palliative care is still beginning.

For peer review only - http://bmjopen.bmj.com/site/about/guidelines.xhtml

- Research took place in medical schools that do not have specialized palliative care placement.
- Observational study could determine if palliative care teaching inserted in non-specialized placements have effects on students' thanatophobia and self-efficacy in palliative care.
- Investigate this topic could bring new evidence on palliative care teaching for medical students in countries with less developed palliative care.

Keywords: Medical Education, Undergraduate; Care, Palliative; Self-assessment; Self-Efficacy; Teaching & Learning;

Introduction

Medicine has made significant advances in improving healthcare outcomes during the last century. While life expectancy has increased in many countries, chronic and degenerative diseases have become the leading causes of death (1,2). To deal with this new epidemiological context, doctors need to become competent to deliver palliative and end of life care (2–4). To prepare doctors to cope with this new reality of practice, medical educators have the responsibility to develop pedagogical strategies to guide medical students towards becoming competent in supporting the psychological, social and spiritual needs of dying patients and their families.

The social cognitive theory suggests that individuals learn and change behaviour through intra-personal motivational constructs, known as 'self-efficacy' and 'outcome expectancy.' "Self-efficacy" relates to the confidence an individual (in this case, medical students) has in their capacity in performing a specific task/behaviour. Students' knowledge, skills, and previous experience, including the observation of other's performance, modulates their self-efficacy (5). Outcome expectancy refers to the perceived consequence of the target behaviour, together with the aggregate value this

For peer review only

behaviour brings to the student. A 'positive' performance is more likely when students' self-efficacy is higher, and the expected outcome is rewarding (5). Thus, appropriate training should not only increase students' knowledge, but also strengthen their self-confidence in achieving their objectives (self-efficacy), while showing the value of the desirable actions (outcome expectancies)(5,6). Social cognitive theory has been employed in studies to assess educational interventions, particularly where practical assessment of the development of knowledge, skills, and attitudes are difficult to apply efficiently at scale (7,8). Consequently, based on this theory, evaluating the design of learning activities should incorporate the measurement of self-efficacy and outcome expectancy.

The Self-efficacy in Palliative Care (SEPC) and the Thanatophobia (TS) scales were developed to evaluate student's self-efficacy and outcome expectancies in palliative care (PC) (6,9,10). The SEPC covers core aspects of doctors' expected behaviours in PC: effective communication with the patient and family; assessment and management of patient's symptoms and needs; and multidisciplinary work within a healthcare team (9). Thanatophobia (literally 'fear of death') relates to healthcare professionals' avoidance of terminally ill patients (11). Lower levels of thanatophobia are considered desirable when healthcare professionals are providing end-of-life care; therefore, the TS scale may be used to measure the outcomes expectancies related to PC education (9,11). Both scales (SEPC and TS) were validated to measure the impact of medical training in PC learning (9).

Although medical schools are recommended to include palliative care in their curricula, the teaching and assessment strategies are heterogenous, and many schools do not have access to PC specialized services for students' placements (12,13). Examples of specialized services are hospices, wards, and outpatients' clinics run by palliative medicine specialists. While in Europe and other countries the palliative care have wider offer and students training are based in specialized services, in Latin America that is not the case (14,15). For example, in Brazil, there are less than 200 specialized

For peer review only - http://bmjopen.bmj.com/site/about/guidelines.xhtml

PC services, and many are not linked to medical schools (16). In practical terms, most of medical students in Brazil do not have access to specialized training settings, which is the case of the medical schools in our research (17). In Brazilian medical school, the overloaded curricula and the lack of specialized palliative care teachers hinder palliative care undergraduate education (13).Therefore, we explore in this article whether a focus on palliative care topics during non-specialized clinical rotations without the active participation of PC specialists may offer efficient learning opportunities for senior medical students and bridge a gap within existing curricula.

Engagement and learning within a specialized practice may be considered ideal for training purposes. However, effective learning is mostly related to how any given scenario is modulated by students and supervisors to optimize the learning process through different lenses (18,19). Among these lenses, the Kolb's cycle offers a compelling insight into understanding the learning process triggered by practical experiences (19). Kolb's cycle stresses the importance of integrating the practical experience with the abstract conceptualization of this experience. Therefore, the learning process occurs through consecutive cycles of lived experiences, reflexive observations, and active planning of the next experimentations supported by appropriate supervision and feedback, in which students integrate, modulate and potentialize their own leaning process (18). Thus, we hypothesize that non-specialized centers can provide a proper learning environment to offer meaningful and productive clinical experiences, which could be capable of grounding the improvement of self-efficacy in PC and the decreasing of thanatophobia levels (19).

To verify our hypothesis, we assessed PC training outcomes in two different medical schools in Brazil. The first medical school offers a clinical rotation with direct contact with critical care and dying patients and opportunities to reflect and discuss the care for dying patients are inserted during the placement. The clinical supervisors within this first school (MS1), although not specialized in PC, accept end-of-life care as part of their professional and educational role as general internal medicine doctors. The

For peer review only - http://bmjopen.bmj.com/site/about/guidelines.xhtml

second medical school (MS2) offers a shorter placement in Anaesthesiology rotation which includes an outpatient service for PC and chronic pain patients and a homecare service to vulnerable patients who have a low performance-status. In the MS2 context, medical students have the opportunity to discuss palliative care topics, such as pain and symptoms management In this longitudinal study, we aimed to measure medical students' self-efficacy in PC and thanatophobia levels, before and after those two different rotations in the aforementioned medical schools. Our hypothesis is that non-specialized rotations are capable of nurturing self-efficacy in PC and decreasing Thanatophobia levels in undergraduate medical students.

Methods

Context

The undergraduate medical course in Brazil is six years long. National guidelines for curriculum development demand the integration of learning activities with healthcare services throughout the training. In general, during the first two years, students attend classroom-based teaching, with a combination of lectures and small group discussions. Besides the classroom activities, students have regular placements in primary care and hospital facilities to be introduced to the logic of patient care. During years three and four, students are trained in history taking, physical examination and clinical reasoning, and they are involved in the longitudinal care of patients in primary care facilities. During the last two years, students undertake an internship, where they rotate in diverse clinical placements such as internal medicine, paediatrics, primary care, gynaecology, surgery, critical care, and medical emergencies. In these clinical rotations, students work under supervision and take direct responsibility for patient care in clinical and surgical wards, outpatient clinics, and emergency departments. After concluding their undergraduate studies, they receive full registration to practice medicine and are allowed to perform independently, even without resident training. Many new doctors start working at

For peer review only - http://bmjopen.bmj.com/site/about/guidelines.xhtml

emergency departments and primary care facilities, despite their lack of postgraduate training.

Design, Setting and Participants

We conducted a longitudinal observational study with two cohorts of Year 6 medical students, from two separate Medical Schools, in 2017. Both medical schools are ranked in the top 10 within Brazil (20,21). Below we briefly describe some aspects of the rotations and details of MS1 and MS2 placements are in Appendix section.

Medical School 1 (MS1): final year students undertake an 8-week mandatory emergency and critical care placement, with 14 students per group. The first group took part in the research during the beginning of the final year, in January 2017, and the last one in September 2017. During this rotation, students engage in clinical activities for 11 hours daily, under the direct supervision of clinical teachers, and experience the care of chronically ill and dying patients. It is hard to quantify exactly how many dying patients the students have contact during the rotation. However, as the rotation take place in a non-surgical ICU, many patients have chronic diseases such as COPD, heart failure, metastatic cancer etc. This ICU has 28 beds and a mortality rate of 30%. Unfortunately, in our context is still common that terminal patients (even those that should be considered for exclusive palliative care) receive their end-of-life care in the ICU or emergency room, as we lack palliative care services in the health network. Considering that, most of MS1 students are involved in caring for patients and assist them in the end of life and almost all testify the deaths of some of the patients during the rotation period. The clinical supervisors decided to include flexible PC discussions in bedside rounds due to the opportunities provided by this placement. Conversely, the supervisors provide formally structured discussions about end-of-life ethics in multi-professional meetings. Also, students have access to training sessions with simulated patients to develop communication skills (including breaking bad news) and emotional competence (22,23).

For peer review only - http://bmjopen.bmj.com/site/about/guidelines.xhtml

As such, students have several opportunities to acquire the knowledge, skills, and attitudes required to care for dying patients, and reflexive learning is stimulated throughout the rotation by the supervisors. Nevertheless, most of the discussions during the rotation are about emergency medicine and critical care, and the activities around the topic of PC are complementary.

Medical School 2 (MS2): final year medical students undertake a 6-week mandatory rotation in Anaesthesiology and pain therapy, in groups of 12 students. The first group answered to this research in February 2017 and the last one in September 2017. Significant part of the training in this rotation is dedicated to Anaesthesia abilities training. Within this period, students also have 32 hours dedicated to specific PC topics and experiences, spread in 3 weeks. Learning opportunities include lectures on PC concepts and pain management, home care visits for PC patients and supervised activities in the outpatient clinic under the supervision of PC consultants. In this placement, students work together with a multi-professional team, which includes social workers, psychologists, nurses, physical therapists, and nutritionists. The students learn how to manage symptoms, work in a multidisciplinary team, and deliver care for PC patients in different settings, such as outpatient clinics and home care. Aside from PC topics, students also engage in learning activities on anaesthesiology.

Procedure

Data were collected from February 2017 to September 2017, when we send the invitation for participation in the study to all the students enrolled in the aforementioned rotations at MS1 and MS2 (80 and 60 students respectively). Two authors, GG and GAMB (supervisor in MS2), personally invited the students during the introductory activities of each rotation in MS1 and MS2, respectively. Both explained to students the research goals, the nature of the instruments that would be applied and guaranteed the anonymity of the participants. Authors also assured that participating in the research would not affect students' grades in that rotation, trying to minimize students' perception of being

For peer review only - http://bmjopen.bmj.com/site/about/guidelines.xhtml

evaluated and its interference in data provided. Students who agreed to participate signed a written informed consent. We collected data from each group of students (MS1 and MS2) in two different time-points of their placements: the first point was at the start of the placement; the second one was during the last week. To report this study we used STROBE cohortreporting guidelines(24).

Questionnaires

Demographic survey: The authors developed a demographic questionnaire to explore students' previous personal and professional experiences related to palliative and end-of-life care (Appendix), as it was expected that past experiences would influence students' learning and performance (25,26).

Self-efficacy in Palliative Care Scale (SEPC) (9): The SEPC measures the self-perceived efficacy in PC by recording students' ratings of their confidence in performing PC related actions, using a 100 mm Visual Analogue Scale. The visual analogue scales range from 'would feel very anxious' to 'would feel very confident.' The point assigned on the visual analog scale is measured and scored from zero up to 100. The 23-item SEPC has three sub-scales of a) Communication (8 items; score ranging from 0 to 800); b) Patient management (8 items; score ranging from 0 to 800); c) Multi-disciplinary team working (7 items; score ranging from 0 to 700).

Thanatophobia Scale (11): The original TS has seven items and assesses attitudes related to the care for dying patients. The scale offers a reliable measure of students' outcome expectancy related to PC (9). Participants rate how much they agree/disagree with expressed negative attitudinal statements, with the final scores ranging from 7 to 49. Higher ratings indicate higher thanatophobia levels.

Both original scale was translated and validated to Brazilian Portuguese before this study (27).

For peer review only - http://bmjopen.bmj.com/site/about/guidelines.xhtml

Ethics

This research was conducted following the Declaration of Helsinki. We assured any student who was uncomfortable with the research enquiring would not feel obliged to participate. Themes related to death could be emotive and/or sensitive; thus, students could contact the researchers to receive support or referral if/as required. The Research Ethics Committee of both medical schools approved the study (58198016.4.0000.5404/2016; 58198016.4.3001.5411/2016).

Patient and Public Involvement

We did not involve patients directly in this study.

Data Analysis

Continuous variables were described by mean, minimal and maximal values, and categorical variables as absolute (n) and relative frequency (%). We analysed the difference between students' pre and post main scores on the scales using t-tests. Effect size was calculated by Cohen's d and used for checking the amplitude of changes in pre- and post-test scores, providing an index to assess the magnitude of the difference detected on t-test results. The effect size was characterized as small, medium, large, or very large and evaluated the magnitude of change despite the sample size. Bonferroni was used to correct the alpha value. Therefore, we used alpha value of 0.125 for all analyses. Data were analysed using IBM-SPSS 21.0 (28).

Results

From the total students invited, two students from each school were excluded due to incomplete data. Thus, we included 78 students from MS1 and 56 students in MS2 (65.0% and 62.2% of last year students, respectively). Students' demographic data and

For peer review only - http://bmjopen.bmj.com/site/about/guidelines.xhtml

previous experiences are summarized in Table 1, which were answered in pre-test data collecting.

Table 1 - Descriptive data of MS1 and MS2

	Medical School 1	Medical School 2
**Sample size**	78	56
**Average age in years (min – max)**	24.8 (22-30)	25.2 (22-34)
**Gender**		
Male	36 (46.2%)	30 (53.6%)
Female	42 (53.8%)	26 (46.4%)
Has anybody that you have been close to died?		
Yes	63 (80.8%)	45 (80.4%)
No	15 (19.2%)	11 (19.6%)
Have you ever witnessed the moment of a person's death?		
Yes	64 (82.1%)	52 (92.9%)
No	14 (17.9%)	4 (7.1%)
During your professional training, have you ever taken care of anybody that eventually died?		
Yes	75 (96.2%)	55 (98.2%)
No	3 (3.8%)	1 (1.8%)

In both schools, students had a significant increase in self-efficacy in all dimensions of the SEPC ($p<0.001$ for all analyses). Students' thanatophobia levels significantly decreased in MS1, but not in MS2, as showed in Table 2 (significant results are highlighted). The effect size of the change in SEPC was larger at MS1 when compared to MS2, except on the SEPC Patient Management subscale, which showed

For peer review only - http://bmjopen.bmj.com/site/about/guidelines.xhtml

a very large effect size in both schools. The effect size magnitude of TS was moderate in MS1.

Table 2 – SEPC and Thanatophobia score means, t-test and effect size at Medical School 1 and 2

Instruments	***Pre-test***	***Post-test***	***Difference Post - Pre***	***t***	***p***	***CI95%***	***Cohen's d***	***Effect Size Magnitude***
**Medical School 1 (n=78)**								
SEPC - Communication	346.50 (145.17)	573.90 (124.47)	227.39 (138.50)	14.50	**<0.001**	**258.62, 196.17**	**1.64**	***Very large***
SEPC - Patient Management	338.38 (112.56)	527.50 (113.55)	189.11 (140.30)	11.90	**<0.001**	**220.75, 157.48**	***1.35***	***Very large***
SEPC - Multidisciplinary Teamwork	361.26 (124.28)	470.35 (105.77)	109.09 (118.86)	8.11	**<0.001**	**135.17, 81.01**	***0.92***	***Large***
TS	21.54 (7.56)	16.54 (7.58)	-5.00 (5.91)	-7.06	**<0.001**	**3.59, 6.41**	***0.79***	***Moderate***
**Medical School 2 (n=56)**								
**SEPC - Communication**	296.41 (147.41)	398.00 (171.64)	101.59 (151.30)	5.02	**<0.001**	**142.11, 61.07**	**0.67**	***Moderate***
**SEPC - Patient Management**	327.86 (133.91)	462.07 (126.55)	134.21 (132.69)	7.57	**<0.001**	**169.75, 98.68**	**1.01**	***Very Large***
**SEPC - Multidisciplinary Teamwork**	388.50 (120.18)	454.96 (120.49)	66.46 (136.82)	3.63	**=0.001**	**103.10, 29.83**	**0.49**	***Small***
**TS**	22.82 (8.77)	21.20 (7.67)	1.62 (5.45)	1.96	<0.05	-0.14, -3.07	0.26	*Small*

Discussion

For peer review only - http://bmjopen.bmj.com/site/about/guidelines.xhtml

In this study, the SEPC and TS measured significant improvements in students' self-efficacy in palliative care and thanatophobia levels, after the placements analysed, except in MS2, in which TS changes were not significant. These results support that non-specialized rotation can provide PC training with effects in students' self-efficacy in PC and thanatophobia. Most of the students in both schools informed some previous experience with dying people, or as a personal experience or during their professional training. This could suggest that previous clinical placements give opportunities for caring of dying patients. Nevertheless, the placements analysed in this study gave to students an additional opportunity to care of dying patients and purposely learn and discuss PC topics.

In both schools, the placements brought improvements in students` self-efficacy in palliative care, in all subscales, as expected after a learning experience that includes PC topics and discussions. Another study with UK medical students had similar results (8). Nevertheless, in the UK study, the effect size magnitude was larger in almost every subscale of SEPC, although the pre-test results were similar. One explanation for this could be the specialized PC placement the UK students had. There are other factors to be considered: time dedicated to PC training, specialized supervisors and services, placement length and the learning environment. In this case, a specialized placement, focused on training PC competencies, could bring development to students' self-efficacy and decrease their thanatophobia. In MS1, a decreasing in TS was observed, but not in MS2. Maybe this is a consequence of the experience in MS1 during the placement is related to more experience with critical and dying patients (see Appendix).

Worldwide, medical educators have devised different strategies for PC training, and there is no 'gold standard' to guide best practices (12,29,30). Although training in specialized PC services could bring larger improvement in SEPC, these services are not common in Brazil and are not an option for training medical students. Including a PC specialized placement in the already busy and crowded medical curriculum poses a challenge for medical educators. The challenge has two edges; one is the scarcity of

For peer review only - http://bmjopen.bmj.com/site/about/guidelines.xhtml

specialized services and professionals to facilitate training, particularly in the developing world (where the PC is still incipient and needs substantial development); the second is the lack of space in the curriculum.

Ultimately, clinicians in most specialties, such as internal medicine, geriatrics, oncology, emergency medicine, intensive care, primary care, paediatrics, and so on, will have PC patients among their caseloads. Although there can often be a lack of PC services, different disciplines and placements should include PC in their curricula and provide PC training for all medical students (31). Teachers could customize different clinical placements to modulate learning possibilities to students considering the resources available as showed in this study medical schools. By doing so, medical educators could ensure a psychological safety environment for learning, including feedback routines and the development of self-efficacy, which we considered as a fundamental aspect in schools we analysed (32). Our study showed that non-specialized rotations, which include specific topics in PC, were capable of improving students` self-efficacy in PC and decreasing their thanatophobia levels.

Therefore, others aspects as learning environment and experience, feedback opportunities and the inclusion of PC as part of clinical caring, could play relevant elements for PC training in this context (19). We believe that this finding is related to opportunities to reflect on real end-of-life cases that were provided for the students during these placements. As anticipated by Kolb`s experiential learning theory, the several occasions to reflect in and on the practice could have nurtured confidence and a readiness to practice PC (19). In fact, the social cognitive theory states that self-efficacy and outcome expectancy are both modulated by the feedback (perceived support) from supervisors, which will eventually, impact on the internalization of the desired behaviour (5). Possibly offering opportunities to experience and reflect, guided by qualified supervision with structured feedback, may be as important as having access to specialized PC centres for the learning of undergraduate students (5,6).

For peer review only - http://bmjopen.bmj.com/site/about/guidelines.xhtml

The challenge posed to medical educators is building up a meaningful learning process in complex workplaces in different clinical rotations, despite the lack of ideal circumstances (18). Clinical teachers need to nurture the learning experience of students embracing the contextual elements, even the negative ones, as teaching assets and not as pitfalls. Students will practice medicine in the real world and need to learn how to deal with the context of the workplace. Those with higher self-efficacy are more likely to reflect on their challenges and experiences to improve performance (5). In this sense, reflection is essential not only to learn but also to feel the urge to change and improve the healthcare delivered to patients (8). Therefore, including structured PC discussions in clinical rotations may offer an opportunity for teachers not only to prepare students to provide proper palliative care but also to open the debate about the relevance of including PC into their future practice.

Strengths and limitations

This study had a consistent methodological approach and involved two different medical schools, with different undergraduate curricula, which included PC training in non-specialized rotations. We demonstrated that, although the two groups of students had different approaches in the sense of learning PC, both groups had improvements in their self-efficacy, even when students did not have access to specialized PC services.

Nevertheless, we should acknowledge some limitations in our study. Firstly, we did not access students' conceptual knowledge of PC, neither the effects of other factors as placement length or learning approaches. In this sense, as we did not control other factors, we cannot conclude if the changes in scales are related to daily placements` clinical situations or to specific teaching approaches. An interventional study with control groups would be more appropriate to verify if and how the different educational approaches could have different impacts on self-efficacy in palliative care and thanatophobia levels.

For peer review only

Secondly, although the students have expressed more confidence in their performance, we cannot conclude that the improvements in SEPC and thanatophobia, observed in this study, will be persistent for the actual medical practice of the students. The proper assessment for this will require further evaluation over the new doctors in their practice. Also, improvements in self-efficacy, in general, can be related to the opportunity given to medical students to reflect on their clinical experiences under the guidance of motivated facilitators (5). This general improvement could have also been extrapolated to Palliative Care competencies (6). Additionally, in MS2, as one of the researchers was supervisor, a socially desirable answer from the participants cannot be excluded.

Furthermore, the findings in this study are limited to two Brazilian medical schools and any generalization should be cautious. Future studies are needed to verify whether our findings will be consistent across different contexts.

Implications for practice and next steps

In a context of an overloaded curriculum and a lack of departments and services of palliative medicine in medical schools, our findings suggest that inserting PC topics in different non-specialized rotations could benefit the development of medical students self-efficacy in PC and reduce their thanatophobia. Thus, a vertical insertion of PC concepts and practices along the course, in different disciplines and rotations, could address PC learning needs for undergraduate students, even without specialized PC services for medical training.

We still need to understand the impact of implementing PC training on clinical rotations on students` declarative and procedural knowledge acquisition. It is also essential to clarify what are the teaching methodologies related to better development of PC competencies in undergraduate medical students.

Conclusion

For peer review only - http://bmjopen.bmj.com/site/about/guidelines.xhtml

Our findings support that non-specialized rotations can include PC basic competencies as part of the training. Medical schools could prepare future doctors to provide PC in any area it is required by embracing palliative care as a core competence, indispensable for becoming a doctor.

Glossary Terms

Palliative care: “approach that improves the quality of life of patients and their families facing the problem associated with life-threating illness, through the prevention and relief of suffering by means of early identification and impeccable assessment and treatment of pain and other problems, physical, psychosocial and spiritual.”(2)

Self-efficacy: “the confidence to carry out the courses of action necessary to accomplish desired goals” (33)**.**

Thanatophobia: death anxiety that caregivers and healthcare professionals may feel when caring for dying patients (11).

Declarations

Ethics approval and consent to participate

This research was conducted following the Declaration of Helsinki. We assured any student who was uncomfortable with the research enquiring would not feel obliged to participate. Themes related to death could be emotive and/or sensitive; thus, students could contact the researchers to receive support or referral if/as required. The Research Ethics Committee of both medical schools approved the study (58198016.4.0000.5404/2016; 58198016.4.3001.5411/2016).

Consent for publication

Not applicable

Availability of data and materials

For peer review only - http://bmjopen.bmj.com/site/about/guidelines.xhtml

The datasets used and analysed during the current study are available from the corresponding author on reasonable request.

Competing interests

GAMB is a supervisor in MS2 and participate in collecting data.

The other authors declare that they have no competing interests.

Funding

This work was supported by CAPES, grant number 88881.188776/2018-01 (PhD sandwich scholarship – Edictal 47/2017) to the first author for analysis and manuscript writing.

Author contributions

GG, SRM and MACF conceived and designed the study. GG and GAMB collected the data. GG, DCF and MACF analysed the data. GG and MACF were the major contributors in manuscript writing. DCF, GAMB and SRM provided meaningful inputs and critical review of the manuscript. All authors read and approved the final manuscript.

Acknowledgements

Palliative Care Institute Liverpool, the University of Liverpool for hosting and supporting the manuscript writing;

Medical students and supervisors involved on data collection;

Author's information

GG, M.Sc., M.D., G.P. is a PhD student at the School of Medical Sciences, UNICAMP, Brazil, in the field of medical education focused on palliative care education;

For peer review only - http://bmjopen.bmj.com/site/about/guidelines.xhtml

DCF, PhD is a young investigator at Department of Medical Psychology and Psychiatry, School of Medical Sciences, UNICAMP, Brazil;

GAMB, MD, MSc, Ph.D. is Associate Professor, Anaesthesiology Department, Botucatu Medical School, Sao Paulo State University - UNESP, Botucatu-SP, Brazil

SRM, PhD is Research Lead at Palliative Care Institute Liverpool, University of Liverpool, Liverpool, UK;

MACF, M.D., PhD is Associate Professor of Emergency Medicine at the School of Medical Sciences, the University of Campinas, Brazil and Senior Researcher at Center for Education Development and Research in Health Professions (CEDAR), University of Groningen, Groningen, the Netherlands;

References

1. Marinho F, de Azeredo Passos VM, Carvalho Malta D, Barboza França E, Abreu DMX, Araújo VEM, et al. Burden of disease in Brazil, 1990–2016: a systematic subnational analysis for the Global Burden of Disease Study 2016. Lancet [Internet]. 2018 [cited 2018 Dec 14];392(10149):760–75. Available from: https://vizhub.

2. Connor SR, Sepulveda Bermedo MC. Global atlas of palliative care at the end of life [Internet]. World Palliative Care Alliance. 2014. 111 p. Available from: http://www.who.int/nmh/Global_Atlas_of_Palliative_Care.pdf

3. Murtagh FE, Bausewein C, Verne J, Groeneveld EI, Kaloki YE, Higginson IJ. How many people need palliative care? A study developing and comparing methods for population-based estimates. Palliat Med [Internet]. 2014 [cited 2019 Mar 6];28(1):49–58. Available from: https://journals.sagepub.com/doi/pdf/10.1177/0269216313489367

4. Etkind SN, Bone AE, Gomes B, Lovell N, Evans CJ, Higginson IJ, et al. How many people will need palliative care in 2040? Past trends, future projections and implications for services. BMC Med [Internet]. 2017 [cited 2019 Mar

For peer review only - http://bmjopen.bmj.com/site/about/guidelines.xhtml

6];15:102. Available from: https://www.ncbi.nlm.nih.gov/pmc/articles/PMC5436458/pdf/12916_2017_Article_860.pdf

5. Artino AR. Academic self-efficacy: from educational theory to instructional practice. Perspect Med Educ [Internet]. 2012 [cited 2019 Feb 20];1(2):76–85. Available from: https://link.springer.com/content/pdf/10.1007%2Fs40037-012-0012-5.pdf

6. Mason SR, Ellershaw J. Preparing for palliative medicine; evaluation of an education programme for fourth year medical undergraduates. Palliat Med [Internet]. 2008 [cited 2019 Feb 11];22:687–92. Available from: https://journals.sagepub.com/doi/pdf/10.1177/0269216308090070

7. Gilissen J, Pivodic L, Wendrich-van Dael A, Cools W, Stichele R Vander, Van den Block L, et al. Nurses' self-efficacy, rather than their knowledge, is associated with their engagement in advence care planning in nurisng homes: a syrvey study. Palliat Med. 2020;34(7):917–34.

8. Mason SR, Ellershaw J. Undergraduate training in palliative medicine: is more necessarily better? Palliat Med [Internet]. 2010 [cited 2019 Jan 29];24(3):306–9. Available from: https://journals.sagepub.com/doi/pdf/10.1177/0269216309351867

9. Mason S, Ellershaw J. Assessing undergraduate palliative care education: Validity and reliability of two scales examining perceived efficacy and outcome expectancies in palliative care. Med Educ. 2004;38(10):1103–10.

10. Neil H, Frey R, Gott M. Instruments used to measure the effectiveness of palliative care education initiatives at the undergraduate level: A systematic literature review. BMJ Support Palliat Care [Internet]. 2013 [cited 2019 Feb 11];3:114–9. Available from: http://www.sherpa.ac.uk/romeo/issn/2045-435X/https://researchspace.auckland.ac.nz/docs/uoa-docs/rights.htm

11. Merrill J, Lorimor R, Thornby J, Woods A. Caring for Terminally ILL Persons:

For peer review only - http://bmjopen.bmj.com/site/about/guidelines.xhtml

Comparative Analysis of Attitudes (Thanatophobia) of Practicing Physicians, Student Nurses, and Medical Students. Psychol Rep. 1998;

12. Fitzpatrick D, Heah R, Patten S, Ward H. Palliative Care in Undergraduate Medical Education-How Far Have We Come? Am J Hosp Palliat Med [Internet]. 2017 [cited 2019 Mar 22];34(8):762–73. Available from: https://journals.sagepub.com/doi/pdf/10.1177/1049909116659737

13. Padilha De Toledo A, Gonçalves Priolli D. Cuidados no Fim da Vida: O Ensino Médico no Brasil (End-of-life Care Education in Brazilian Medical Schools). Rev Bras Educ Med [Internet]. 2012 [cited 2019 Feb 28];36(1):109–17. Available from: http://www.scielo.br/pdf/rbem/v36n1/a15v36n1.pdf

14. Carrasco JM, Lynch TJ, Garralda E, Woitha K, Elsner F, Filbet M, et al. Palliative Care Medical Education in European Universities: A Descriptive Study and Numerical Scoring System Proposal for Assessing Educational Development. J Pain Symptom Manage [Internet]. 2015 Oct 1 [cited 2019 Feb 28];50(4):516-523.e2. Available from: https://www.sciencedirect.com/science/article/pii/S0885392415002419

15. Vindrola-Padros C, Mertnoff R, Lasmarias C, Gómez-Batiste X. Palliative care education in Latin America: A systematic review of training programs for healthcare professionals. Palliat Support Care. 2018;16(1).

16. Análise Situacional e Recomendações para Estruturação de Programas de Cuidados Paliativos no Brasil. São Paulo: Academia Nacional de Cuidados Paliativos; 2018.

17. Oliveira JR de, Ferreira AC, Rezende NA de. Ensino de bioética e cuidados paliativos nas escolas médicas do Brasil. Rev Bras Educ Med [Internet]. 2013 [cited 2018 Dec 12];37(2):285–90. Available from: http://www.scielo.br/pdf/rbem/v37n2/17.pdf

18. Yardley S, Teunissen PW, Dornan T. Experiential learning: AMEE Guide No. 63. Med Teach. 2012 Feb;34(2).

For peer review only - http://bmjopen.bmj.com/site/about/guidelines.xhtml

19. Taylor DCM, Hamdy H. Adult learning theories: Implications for learning and teaching in medical education: AMEE Guide No. 83. Med Teach. 2013 Nov;35(11).

20. Medicina - Ranking de Cursos - Ranking Universitário Folha - 2018 [Internet]. [cited 2019 May 9]. Available from: https://ruf.folha.uol.com.br/2018/ranking-de-cursos/medicina/

21. Universities | Top Universities [Internet]. [cited 2019 May 9]. Available from: https://www.topuniversities.com/universities/country/brazil/subject/medicine

22. Schweller M, Ribeiro DL, Passeri SR, Wanderley JS, Carbalho-Filho MA. Simulated medical consultations with standardized patients: In-depth debriefing based on dealing with emotions. Rev Bras Educ Med [Internet]. 2018 [cited 2019 Jun 13];42(1):82–91. Available from: http://dx.doi.org/10.1590/1981-52712018v42n1RB20160089

23. Schweller M, Costa FO, Antonio MAR, Amaral EM, Carvalho-Filho MA de. The impact of simulated medical consultations in the empathy levels of students at one medical school. Acad Med [Internet]. 2014 [cited 2019 Mar 5];89(4):632–7. Available from: https://www.ncbi.nlm.nih.gov/pmc/articles/PMC4885552/pdf/acm-89-632.pdf

24. von Elm E, Altman D, Egger M, Pocock S, Gotzsche P, Vandenbroucke J. The Strengthening the Reporting of Observational Studies in Epidemiology (STROBE) Statement: guidelines for reporting observational studies. BMJ. 2007;335:805–8.

25. Whyte R, Quince T, Benson J, Wood D, Barclay S. Medical students' experience of personal loss: incidence and implications. BMC Med Educ [Internet]. 2013 [cited 2019 Mar 4];13:36. Available from: http://www.biomedcentral.com/1472-6920/13/36

26. Barclay S, Whyte R, Thiemann P, Benson J, Wood DF, Parker RA, et al. An important but stressful part of their future work: Medical students' attitudes to

For peer review only - http://bmjopen.bmj.com/site/about/guidelines.xhtml

palliative care throughout their course. J Pain Symptom Manage [Internet]. 2015;49(2):231–42. Available from: http://dx.doi.org/10.1016/j.jpainsymman.2014.06.004

27. Gryschek G, Cecilio-Fernandes D, Mason S, de Carvalho-Filho MA. Assessing palliative care education in undergraduate medical students: translation and validation of the Self-Efficacy in Palliative Care and Thanatophobia Scales for Brazilian Portuguese. BMJ Open. 2020;10(6):e034567.

28. IBM Corp. IBM SPSS Statistics for Windows. Armonk, NY: IBM Corp.; 2012.

29. Field D, Wee B. Preparation for palliative care: Teaching about death, dying and bereavement in UK medical schools 2000-2001. Med Educ. 2002;36(6):561–7.

30. Schulz-Quach C, Wenzel-Meyburg U, Fetz K. Can e-learning be used to teach palliative care?-medical students' acceptance, knowledge, and self-estimation of competence in palliative care after elearning. BMC Med Educ [Internet]. 2018 [cited 2019 Mar 22];18(82). Available from: https://doi.org/10.1186/s12909-018-1186-2

31. Lloyd-Williams M, Macleod RDM, Macleod RD. A systematic review of teaching and learning in palliative care within the medical undergraduate curriculum A systematic review of teaching and learning in palliative care within the medical undergraduate curriculum. Med Teach [Internet]. 2004 [cited 2019 Feb 12];26(8):683–90. Available from: https://doi.org/10.1080/01421590400019575

32. Caverzagie KJ, Goldenberg MG, Hall JM. Psychology and learning: The role of the clinical learning environment. Med Teach [Internet]. 2019;41(4):375–9. Available from: https://doi.org/10.1080/0142159X.2019.1567910

33. Klassen RM, Klassen JRL. Self-efficacy beliefs of medical students: a critical review. Perspect Med Educ [Internet]. 2018 [cited 2019 Feb 20];7(2):76–82. Available from: https://doi.org/10.1007/s40037-018-0411-3

For peer review only

Appendix

Survey model/ Portuguese-BR version of SEPC and Thanatophobia Scale	
Original Questions	*English Translation*
Você já perdeu na morte alguém que, segundo a sua percepção, teve um papel importante na sua vida?	**Has anybody that you have been close to died?**
Você já viu alguém morrendo?	**Have you ever witnessed a person's death?**
Durante sua formação profissional, você já participou do cuidado de alguém que acabou morrendo?	**During your professional training, have you ever taken care of anybody that eventually died?**

Medical Schools` Placements

In **Medical School 1**, students rotate in three different clinical environments. In the first 4 weeks, they stay in the Emergency Department. For an international audience, it is important to explain that Emergency Departments in Brazil are chaotic environments that are always working 4-5 times above their capacities. For instance, in medical school 1, the Emergency Department was planned to have 25 patients in observation but end up admitting more than 100. Ideally, patients should stay there for 24 hours maximum, but occasionally they stay there for up to two weeks. Even patient who need intensive care can stay in the Emergency Department for up to 5 days waiting for a vacancy inside the hospital. Students engage actively in the process of care and take direct responsibility for patients, always under the supervision of an experienced physician. In the following 4 weeks, students stay for 2 weeks in an ICU, and 2 weeks in an internal medicine with a high level of complexity, both devoted to patients admitted through the Emergency Department. In both, the internal medicine ward and ICU, in the morning, students are responsible for presenting the case of the patients during the daily clinical round and for organizing and implementing the therapeutic plan that is discussed together with the multi-professional team. On Mondays and Fridays mornings, after the round, students engage in a clinical ethics discussion about one of the patients they are caring for. In the afternoon, on Wednesdays and Thursdays, students engage in simulation activities dedicated to address communication issues and develop emotional competence. Every Friday afternoon, students have discussions about death and dying and palliative care. The structure of the weeks in the mentioned rotations are shown below.

**MS1 – Week Planning**	**Monday**	**Tuesday**	**Wednesday**	**Thursday**	**Friday**
**Weeks 1 to 4 Morning**	Emergency Department	Emergency Department	Emergency Department	Emergency Department	Emergency Department
**Afternoon**	Emergency Department	Emergency Department	Emergency Department	Emergency Department	Emergency Department
**Weeks 4 to 8 Morning**	ICU Clinical Round Ethics Discussion	ICU Clinical Round	ICU Clinical Round	ICU Clinical Round Ethics Discussion	ICU Clinical Round
**Afternoon**	Lectures	Lectures	Simulation Sessions	Simulation Sessions	Discussions about death and dying

For peer review only

Medical School 1 – Placement Summary

**Placement**	Emergency and Critical Care Medicine
**Length**	8 weeks
**Program hours**	440 hours
**Learning Outcomes related to Palliative Care**	- Identify terminal patients and understand the moment to shift from a predominant curative approach to a predominant palliative approach.<br>- Provide end-of-life care to terminal patients<br>- Understand the concepts of ortothanasia and dysthanasia<br>- Understand and apply the ethical principles of surrogate decision-making<br>- Understand and apply the principles of interprofessional collaboration in the context of end-of-life care<br>- Understand how the different cultural perspectives related to death and dying impact on end-of-life care<br>- Understand how our own perspectives on death and dying may interfere with the end-of-life care that we provide to our patients<br>- Develop patient-centered communication based on presence, improvisation, and empathy.
**Groups size**	14 students
**Learning activities**	- Daily rounds to: emergency department and critical care wards;<br>- Case discussion sessions;<br>- High fidelity simulation sessions: emergency room and consultation scenarios;<br>- Point-of-care ultrasound classes and bedside training;<br>- Remote learning: digital educational plataform Moodle;<br>- Mechanical ventilation classes;<br>- Palliative care classes;<br>- Palliative care ethics discussions;
**Palliative care topics**	- Breaking bad news;<br>- End-of-life ethics;<br>- Thanatology;
**Total Supervisors**	All Medical doctors<br>- Emergency department: 8<br>- Internal Medicine Ward: 2<br>- Intensive Care Unit: 2
**Supervisors experience in Medical Education**	The course coordinator is a researcher in medical education, and 2 of the eight supervisors have a Ph.D. in medical education focused on developing new active learning strategies. All the supervisors have consistently participated in medical education congresses, presenting both research and educational innovations.
**Supervisors experience in Palliative Care**	Lecturer in Palliative care topics<br>End of life care ethics discussions in weekly basis;

For peer review only

**Educational Methods**	- Daily wards rounds;<br>- Blended methods: classes and remote learning;<br>- Bedside teaching;<br>- Simulation sessions;<br>- Clinical case discussions;
**Learning activities related to PC and end-of-life care**	- Clinical simulation targeting breaking bad news, communication skills, and emotional competence (4 sessions of 4h each);<br>- End of Life Ethics discussions (4 sessions of 1 hour each);
**Teaching highlights**	- Simulation and feedback<br>- Reflexive learning<br>- Case discussion of complex and critical patients
**Multidisciplinary team coworking**	Coworking close to other medical specialties: psychiatry, neurology, orthopedics and traumatology
**Supervisors Feedback routine**	Daily feedback in wards rounds and other learning activities, such as Moodle and simulation sessions.<br>Grades mailing in the end of the placement with all evaluation results
**Evaluation**	Multidimensional evaluation<br>- Two theorical classic tests: multiple choice and open questions<br>- OSCE: clinical scenarios based in placement daily situations<br>- Supervisors, peers and self-evaluation on attitudes and behavior

In **Medical School 2** the Anesthesiology and Pain Medicine placement have a total of 280 hours of activities. About 140 hours of this workload is related to palliative care topics along pain medicine learning activities. The group of students are divided into sub-groups composed of 2 students to palliative care activities, which are performed during home visits, in wards and in outpatient clinics, assisting patients under palliative care. All these clinical-practical activities take place in multiprofessional settings with the presence of nurses, social workers and other professionals. At the same time there are theoretical activities in which subjects such as the treatment of acute and chronic pain, bases of palliative care and health communication are addressed, with an emphasis on bad news.

**MS2 – Week Planning**	**Monday**	**Tuesday**	**Wednesday**	**Thursday**	**Friday**
**Weeks 1 to 3 Morning**	Anesthesiology department	Anesthesiology department	Anesthesiology department	Anesthesiology department	Anesthesiology department
**Afternoon**	Anesthesiology department	Anesthesiology department	Anesthesiology department	Anesthesiology department	Anesthesiology department
**Weeks 3 to 6 Morning**	Pain and Palliative Care Outpatient Clinic	Pain and Palliative Care Outpatient Clinic	Pain and Palliative Care Outpatient Clinic	Pain and Palliative Care Outpatient Clinic	Pain and Palliative Care Outpatient Clinic
**Afternoon**	Lecture	Lecture	Clinical case discussion	Pain and Palliative Care Outpatient Clinic	Homecare

For peer review only

Medical School 2 – Placement Summary

**Clinical placement**	Anesthesiology and Pain Medicine
**Length**	6 weeks
**Total program hours**	280 hours
**Learning Outcomes related to Palliative Care**	- Identify patients who can benefit from palliative care<br>- Understand the concepts of palliative care<br>- Understand the importance of adequate communication in health and symptoms management<br>- Evaluate and manage acute and chronic pain
**Groups size**	12 students
**Learning activities**	- Traditional and interactive lectures using Kahoot (game-based learning platform)<br>- Patient care in outpatient clinic, in the hospital yards and home care<br>- Clinical cases discussion
**Palliative care topics**	- Palliative care concepts<br>- Communication in palliative care<br>- Pain and symptom management
**Total Supervisors**	- Medical doctors: 2<br>- Registered Nurses: 2
**Supervisors experience in Medical Education**	Medical Doctors are PhD, working as clinical teachers at the medical school for more than 10 years
**Supervisors experience in Palliative Care**	Medical Doctors are board-certificated palliative care specialists<br>Registered Nurses have no specific training
**Educational Methods**	- Lectures<br>- Clinical case discussions<br>- Training procedures skills<br>- Clinical exposure to palliative patient care
**Learning activities related to PC and end-of-life care**	- PC lectures (8h): concepts, communications skills, symptoms management<br>- Clinical discussions of outpatient patients (20h)<br>- Homecare visits (4h)
**Teaching highlights**	- Lectures<br>- Case discussions of chronic patients<br>- Working in a multi-professional healthcare team specialized in Palliative Care
**Multidisciplinary team coworking**	Discussion of clinical home care cases with the participation of registered nurse, social worker, psychologist and nutritionist. Physical therapists are available under referral
**Supervisors Feedback routine**	During daily practical activities
**Evaluation**	Classical style tests and clinical skill practical evaluation using simulated setting activities

For peer review only

Reporting checklist for cohort study.

Based on the STROBE cohort guidelines.

Instructions to authors

Complete this checklist by entering the page numbers from your manuscript where readers will find each of the items listed below.

Your article may not currently address all the items on the checklist. Please modify your text to include the missing information. If you are certain that an item does not apply, please write "n/a" and provide a short explanation.

Upload your completed checklist as an extra file when you submit to a journal.

In your methods section, say that you used the STROBE cohortreporting guidelines, and cite them as:

von Elm E, Altman DG, Egger M, Pocock SJ, Gotzsche PC, Vandenbroucke JP. The Strengthening the Reporting of Observational Studies in Epidemiology (STROBE) Statement: guidelines for reporting observational studies.

		Reporting Item	Page Number
**Title and abstract**			
Title	#1a	Indicate the study’s design with a commonly used term in the title or the abstract	1
Abstract	#1b	Provide in the abstract an informative and balanced summary of what was done and what was found	2
**Introduction**			
Background / rationale	#2	Explain the scientific background and rationale for the investigation being reported	3-5
Objectives	#3	State specific objectives, including any prespecified hypotheses	6
**Methods**			

For peer review only

Study design	#4	Present key elements of study design early in the paper	7
Setting	#5	Describe the setting, locations, and relevant dates, including periods of recruitment, exposure, follow-up, and data collection	7-10
Eligibility criteria	#6a	Give the eligibility criteria, and the sources and methods of selection of participants. Describe methods of follow-up.	6-8
Eligibility criteria	#6b	For matched studies, give matching criteria and number of exposed and unexposed	n/a
Variables	#7	Clearly define all outcomes, exposures, predictors, potential confounders, and effect modifiers. Give diagnostic criteria, if applicable	9
Data sources / measurement	#8	For each variable of interest give sources of data and details of methods of assessment (measurement). Describe comparability of assessment methods if there is more than one group. Give information separately for for exposed and unexposed groups if applicable.	9
Bias	#9	Describe any efforts to address potential sources of bias	8, 9
Study size	#10	Explain how the study size was arrived at	8
Quantitative variables	#11	Explain how quantitative variables were handled in the analyses. If applicable, describe which groupings were chosen, and why	9
Statistical methods	#12a	Describe all statistical methods, including those used to control for confounding	10
Statistical methods	#12b	Describe any methods used to examine subgroups and interactions	10
Statistical methods	#12c	Explain how missing data were addressed	10
Statistical methods	#12d	If applicable, explain how loss to follow-up was addressed	10
Statistical methods	#12e	Describe any sensitivity analyses	n/a

Results

For peer review only

Participants	#13a	Report numbers of individuals at each stage of study—eg numbers potentially eligible, examined for eligibility, confirmed eligible, included in the study, completing follow-up, and analysed. Give information separately for for exposed and unexposed groups if applicable.	10
Participants	#13b	Give reasons for non-participation at each stage	10
Participants	#13c	Consider use of a flow diagram	n/a
Descriptive data	#14a	Give characteristics of study participants (eg demographic, clinical, social) and information on exposures and potential confounders. Give information separately for exposed and unexposed groups if applicable.	10, 11
Descriptive data	#14b	Indicate number of participants with missing data for each variable of interest	10
Descriptive data	#14c	Summarise follow-up time (eg, average and total amount)	n/a
Outcome data	#15	Report numbers of outcome events or summary measures over time. Give information separately for exposed and unexposed groups if applicable.	11-12
Main results	#16a	Give unadjusted estimates and, if applicable, confounder-adjusted estimates and their precision (eg, 95% confidence interval). Make clear which confounders were adjusted for and why they were included	11-12
Main results	#16b	Report category boundaries when continuous variables were categorized	11-12
Main results	#16c	If relevant, consider translating estimates of relative risk into absolute risk for a meaningful time period	n/a
Other analyses	#17	Report other analyses done—e.g., analyses of subgroups and interactions, and sensitivity analyses	n/a
**Discussion**			
Key results	#18	Summarise key results with reference to study objectives	12,13
Limitations	#19	Discuss limitations of the study, taking into account sources of potential bias or imprecision. Discuss both direction and magnitude of any potential bias.	15

For peer review only

Interpretation	#20	Give a cautious overall interpretation considering objectives, limitations, multiplicity of analyses, results from similar studies, and other relevant evidence.	13-15
Generalisability	#21	Discuss the generalisability (external validity) of the study results	16
**Other Information**			
Funding	#22	Give the source of funding and the role of the funders for the present study and, if applicable, for the original study on which the present article is based	18

The STROBE checklist is distributed under the terms of the Creative Commons Attribution License CC-BY. This checklist was completed on 02 August 2020 using https://www.goodreports.org/, a tool made by the EQUATOR Network in collaboration with Penelope.ai

For peer review only

BMJ Open

Examining the effect of non-specialized clinical rotations upon medical students' Thanatophobia and Self-efficacy in Palliative Care: a prospective observational study in two medical schools

Journal:	*BMJ Open*
Manuscript ID	bmjopen-2020-041144.R2
Article Type:	Original research
Date Submitted by the Author:	30-Sep-2020
Complete List of Authors:	Gryschek, Guilherme; State University of Campinas Faculty of Medical Sciences, Internal Medicine<br>Cecilio-Fernandes, Dario; State University of Campinas Faculty of Medical Sciences,<br>Barros, Guilherme; Universidade Estadual Paulista Júlio de Mesquita Filho Câmpus de Botucatu Faculdade de Medicina, Department of Anesthesiology<br>Mason, Stephen; University of Liverpool Marie Curie Palliative Care Institute Liverpool<br>de Carvalho-Filho, Marco Antonio; University Medical Centre Groningen, CEDAR - Center for Educational Development and Research in Health Sciences; University of Minho, Life and Health Sciences Research Institute
**Primary Subject Heading**:	Medical education and training
Secondary Subject Heading:	Palliative care, Medical education and training
Keywords:	MEDICAL EDUCATION & TRAINING, PALLIATIVE CARE, Adult palliative care < PALLIATIVE CARE

SCHOLARONE™ Manuscripts

I, the Submitting Author has the right to grant and does grant on behalf of all authors of the Work (as defined in the below author licence), an exclusive licence and/or a non-exclusive licence for contributions from authors who are: i) UK Crown employees; ii) where BMJ has agreed a CC-BY licence shall apply, and/or iii) in accordance with the terms applicable for US Federal Government officers or employees acting as part of their official duties; on a worldwide, perpetual, irrevocable, royalty-free basis to BMJ Publishing Group Ltd ("BMJ") its licensees and where the relevant Journal is co-owned by BMJ to the co-owners of the Journal, to publish the Work in this journal and any other BMJ products and to exploit all rights, as set out in our licence.

The Submitting Author accepts and understands that any supply made under these terms is made by BMJ to the Submitting Author unless you are acting as an employee on behalf of your employer or a postgraduate student of an affiliated institution which is paying any applicable article publishing charge ("APC") for Open Access articles. Where the Submitting Author wishes to make the Work available on an Open Access basis (and intends to pay the relevant APC), the terms of reuse of such Open Access shall be governed by a Creative Commons licence – details of these licences and which Creative Commons licence will apply to this Work are set out in our licence referred to above.

Other than as permitted in any relevant BMJ Author's Self Archiving Policies, I confirm this Work has not been accepted for publication elsewhere, is not being considered for publication elsewhere and does not duplicate material already published. I confirm all authors consent to publication of this Work and authorise the granting of this licence.

For peer review only

Title: *"Examining the effect of non-specialized clinical rotations upon medical students' Thanatophobia and Self-efficacy in Palliative Care: a prospective observational study in two medical schools"*

Running Title: Palliative care education and medical students

Word count: 3937

Authors:

- Guilherme Gryschek, MD, MSc is a Ph.D. student at School of Medical Sciences – University of Campinas (UNICAMP), Campinas-SP, Brazil; ggryschek@gmail.com

- Dario Cecilio-Fernandes, Ph.D., is a researcher the Department of Medical Psychology and Psychiatry, School of Medical Sciences – University of Campinas (UNICAMP), Campinas-SP, Brazil; dario.fernandes@gmail.com

- Guilherme Antonio Moreira de Barros, MD, MSc, Ph.D. is Associate Professor, Anaesthesiology Department, Botucatu Medical School, Sao Paulo State University - UNESP, Botucatu-SP, Brazil; guilherme.am.barros@unesp.br

- Stephen R Mason, PGCHE, Ph.D., Research Lead Palliative Care Institute Liverpool, University of Liverpool, UK; stephen.mason@liverpool.ac.uk

- Marco Antonio de Carvalho Filho (corresponding author), MD, Ph.D., Associate Professor, Life and Health Sciences Research Institute (ICVS), Medical School, University of Minho; and Senior Researcher, Center for Educational Development and Research in Health Professions (CEDAR), research group LEARN, the University of Groningen, The Netherlands; m.a.de.carvalho.filho@umcg.nl. Address: Antonius Deusinglaan 1 9713AV, Groningen, The Netherlands

For peer review only

For peer review only - http://bmjopen.bmj.com/site/about/guidelines.xhtml

Abstract

Introduction: Including palliative care (PC) in overloaded medical curricula is a challenge, especially where there is a lack of PC specialists. We hypothesized that non-specialized rotations could provide meaningful PC learning when there are enough clinical experiences, with adequate feedback.

Objective: observe the effects of including PC topics in non-specialized placements for undergraduate medical students in two different medical schools.

Design: observational prospective study;

Setting: Medical schools in Brazil;

Participants: 134 sixth-year medical students of two medical schools;

Methods: This was a longitudinal study that observed the development of Self-efficacy in Palliative Care and Thanatophobia in sixth-year medical students in different non-specialized clinical rotations in two Brazilian medical schools (MS1 and MS2). We enrolled 78 students in MS1 during the Emergency and Critical Care rotation and 56 students in MS2 during the rotation in Anaesthesiology. Both schools provide PC discussions with different learning environment and approaches.

Primary Outcomes: Self-efficacy in Palliative Care (SEPC) and Thanatophobia (TS) scales were used to assess students at the beginning and the end of the rotations.

Results: In both schools' students had an increase in SEPC and a decrease in TS scores.

Conclusion: Non-specialized rotations that consider PC competencies as core aspects of being a doctor can be effective to develop self-efficacy in PC and decrease Thanatophobia levels.

Article Summary: Strengths and limitations of this study

- This study access students' self-efficacy in palliative care of two Brazilian medical schools, where palliative care is still beginning.

For peer review only - http://bmjopen.bmj.com/site/about/guidelines.xhtml

- Research took place in medical schools that do not have specialized palliative care placement.
- Observational study could determine if palliative care teaching inserted in non-specialized placements have effects on students' thanatophobia and self-efficacy in palliative care.
- Investigate this topic could bring new evidence on palliative care teaching for medical students in countries with less developed palliative care.

Keywords: Medical Education, Undergraduate; Care, Palliative; Self-assessment; Self-Efficacy; Teaching & Learning;

Introduction

Medicine has made significant advances in improving healthcare outcomes during the last century. While life expectancy has increased in many countries, chronic and degenerative diseases have become the leading causes of death (1,2). To deal with this new epidemiological context, doctors need to become competent to deliver palliative and end of life care (2–4). To prepare doctors to cope with this new reality of practice, medical educators have the responsibility to develop pedagogical strategies to guide medical students towards becoming competent in supporting the psychological, social and spiritual needs of dying patients and their families.

The social cognitive theory suggests that individuals learn and change behaviour through intra-personal motivational constructs, known as 'self-efficacy' and 'outcome expectancy.' "Self-efficacy" relates to the confidence an individual (in this case, medical students) has in their capacity in performing a specific task/behaviour. Students' knowledge, skills, and previous experience, including the observation of other's performance, modulates their self-efficacy (5). "Outcome expectancy" refers to the perceived consequence of the target behaviour, together with the aggregate value this

For peer review only - http://bmjopen.bmj.com/site/about/guidelines.xhtml

For peer review only

behaviour brings to the student (5,6). A 'positive' performance is more likely when students' self-efficacy is higher, and the expected outcome is rewarding (5). Thus, appropriate training should not only increase students' knowledge, but also strengthen their self-confidence in achieving their objectives (self-efficacy), while showing the value of the desirable actions (outcome expectancies)(5,7). Social cognitive theory has been employed in studies to assess educational interventions, particularly where practical assessment of the development of knowledge, skills, and attitudes are difficult to apply efficiently at scale (8,9). Consequently, based on this theory, evaluating the design of learning activities should incorporate the measurement of self-efficacy and outcome expectancy.

The Self-efficacy in Palliative Care (SEPC) and the Thanatophobia (TS) scales were developed to evaluate student's self-efficacy and outcome expectancies in palliative care (PC) (6,7,10). The SEPC covers core aspects of doctors' expected behaviours in PC: effective communication with the patient and family; assessment and management of patient's symptoms and needs; and multidisciplinary work within a healthcare team (6). Thanatophobia (literally 'fear of death') relates to healthcare professionals' avoidance of terminally ill patients (11). Lower levels of thanatophobia are considered desirable when healthcare professionals are providing end-of-life care; therefore, the TS scale may be used to measure the outcomes expectancies related to PC education (6,11). Both scales (SEPC and TS) were validated to measure the impact of medical training in PC learning (6).

Although medical schools are recommended to include palliative care in their curricula, the teaching and assessment strategies are heterogenous, and many schools do not have access to PC specialized services for students' placements (12,13). Examples of specialized services are hospices, wards, and outpatients' clinics run by palliative medicine specialists. While in Europe and other countries the palliative care have wider offer and students training are based in specialized services, in Latin America that is not the case (14,15). For example, in Brazil, there are less than 200 specialized

For peer review only - http://bmjopen.bmj.com/site/about/guidelines.xhtml

PC services, and many are not linked to medical schools (16). In practical terms, most of medical students in Brazil do not have access to specialized training settings, which is the case of the medical schools in our research (17). In Brazilian medical school, the overloaded curricula and the lack of specialized palliative care teachers hinder palliative care undergraduate education (13).Therefore, we explore in this article whether a focus on palliative care topics during non-specialized clinical rotations without the active participation of PC specialists may offer efficient learning opportunities for senior medical students and bridge a gap within existing curricula.

Engagement and learning within a specialized practice may be considered ideal for training purposes. However, effective learning is mostly related to how any given scenario is modulated by students and supervisors to optimize the learning process through different lenses (18,19). Among these lenses, the Kolb's cycle offers a compelling insight into understanding the learning process triggered by practical experiences (19). Kolb's cycle stresses the importance of integrating the practical experience with the abstract conceptualization of this experience. Therefore, the learning process occurs through consecutive cycles of lived experiences, reflexive observations, and active planning of the next experimentations supported by appropriate supervision and feedback, in which students integrate, modulate and potentialize their own leaning process (18). Thus, we hypothesize that non-specialized placements can provide a proper learning environment to offer meaningful and productive clinical experiences, which could be capable of grounding the improvement of self-efficacy in PC and the decreasing of thanatophobia levels (19).

To verify our hypothesis, we assessed PC training outcomes in two different medical schools in Brazil. The first medical school offers a clinical rotation with direct contact with critical care and dying patients and opportunities to reflect and discuss the care for dying patients are inserted during the placement. The clinical supervisors within this first school (MS1), although not specialized in PC, accept end-of-life care as part of their professional and educational role as general internal medicine doctors. The

For peer review only - http://bmjopen.bmj.com/site/about/guidelines.xhtml

second medical school (MS2) offers a shorter placement in Anaesthesiology rotation which includes an outpatient service for PC and chronic pain patients and a homecare service to vulnerable patients who have a low performance-status. In the MS2 context, medical students have the opportunity to discuss palliative care topics, such as pain and symptoms management In this longitudinal study, we aimed to measure medical students' self-efficacy in PC and thanatophobia levels, before and after those two different rotations in the aforementioned medical schools. Our hypothesis is that non-specialized rotations are capable of nurturing self-efficacy in PC and decreasing Thanatophobia levels in undergraduate medical students.

Methods

Context

The undergraduate medical course in Brazil is six years long. National guidelines for curriculum development demand the integration of learning activities with healthcare services throughout the training. In general, during the first two years, students attend classroom-based teaching, with a combination of lectures and small group discussions. Besides the classroom activities, students have regular placements in primary care and hospital facilities to be introduced to the logic of patient care. During years three and four, students are trained in history taking, physical examination and clinical reasoning, and they are involved in the longitudinal care of patients in primary care facilities. During the last two years, students undertake an internship, where they rotate in diverse clinical placements such as internal medicine, paediatrics, primary care, gynaecology, surgery, critical care, and medical emergencies. In these clinical rotations, students work under supervision and take direct responsibility for patient care in clinical and surgical wards, outpatient clinics, and emergency departments. After concluding their undergraduate studies, they receive full registration to practice medicine and are allowed to perform independently, even without resident training. Many new doctors start working at

For peer review only - http://bmjopen.bmj.com/site/about/guidelines.xhtml

emergency departments and primary care facilities, despite their lack of postgraduate training.

Design, Setting and Participants

We conducted a longitudinal observational study with two cohorts of Year 6 medical students, from two separate Medical Schools, in 2017. Both medical schools are ranked in the top 10 within Brazil (20,21). Below we briefly describe some aspects of the rotations and details of MS1 and MS2 placements are in Appendix section.

Medical School 1 (MS1): final year students undertake an 8-week mandatory emergency and critical care placement, with 14 students per group. The first group took part in the research during the beginning of the final year, in January 2017, and the last one in September 2017. During this rotation, students engage in clinical activities for 11 hours daily, under the direct supervision of clinical teachers, and experience the care of chronically ill and dying patients. It is hard to quantify exactly how many dying patients the students have contact during the rotation. However, as the rotation take place in a non-surgical ICU, many patients have chronic diseases such as COPD, heart failure, metastatic cancer etc. This ICU has 28 beds and a mortality rate of 30%. Unfortunately, in our context is still common that terminal patients (even those that should be considered for exclusive palliative care) receive their end-of-life care in the ICU or emergency room, as we lack palliative care services in the health network. Considering that, most of MS1 students are involved in caring for patients and assist them in the end of life and almost all testify the deaths of some of the patients during the rotation period. The clinical supervisors decided to include flexible PC discussions in bedside rounds due to the opportunities provided by this placement. Conversely, the supervisors provide formally structured discussions about end-of-life ethics in multi-professional meetings. Also, students have access to training sessions with simulated patients to develop communication skills (including breaking bad news) and emotional competence (22,23).

For peer review only - http://bmjopen.bmj.com/site/about/guidelines.xhtml

As such, students have several opportunities to acquire the knowledge, skills, and attitudes required to care for dying patients, and reflexive learning is stimulated throughout the rotation by the supervisors. Nevertheless, most of the discussions during the rotation are about emergency medicine and critical care, and the activities around the topic of PC are complementary.

Medical School 2 (MS2): final year medical students undertake a 6-week mandatory rotation in Anaesthesiology and pain therapy, in groups of 12 students. The first group answered to this research in February 2017 and the last one in September 2017. Significant part of the training in this rotation is dedicated to Anaesthesia abilities training. Within this period, students also have 32 hours dedicated to specific PC topics and experiences, spread in 3 weeks. Learning opportunities include lectures on PC concepts and pain management, home care visits for PC patients and supervised activities in the outpatient clinic under the supervision of PC consultants. In this placement, students work together with a multi-professional team, which includes social workers, psychologists, nurses, physical therapists, and nutritionists. The students learn how to manage symptoms, work in a multidisciplinary team, and deliver care for PC patients in different settings, such as outpatient clinics and home care. Aside from PC topics, students also engage in learning activities on anaesthesiology.

Procedure

Data were collected from February 2017 to September 2017, when we send the invitation for participation in the study to all the students enrolled in the aforementiovned rotations at MS1 and MS2 (80 and 60 students respectively). Two authors, GG and GAMB (supervisor in MS2), personally invited the students during the introductory activities of each rotation in MS1 and MS2, respectively. Both explained to students the research goals, the nature of the instruments that would be applied and guaranteed the anonymity of the participants. Authors also assured that participating in the research would not affect students' grades in that rotation, trying to minimize students' perception of being

For peer review only - http://bmjopen.bmj.com/site/about/guidelines.xhtml

evaluated and its interference in data provided. Students who agreed to participate signed a written informed consent. We collected data from each group of students (MS1 and MS2) in two different time-points of their placements: the first point was at the start of the placement; the second one was during the last week. To report this study we used STROBE cohort reporting guidelines (24).

Questionnaires

Demographic survey: The authors developed a demographic questionnaire to explore students' previous personal and professional experiences related to palliative and end-of-life care (Appendix), as it was expected that past experiences would influence students' learning and performance (25,26).

Self-efficacy in Palliative Care Scale (SEPC) (6): The SEPC measures the self-perceived efficacy in PC by recording students' ratings of their confidence in performing PC related actions, using a 100 mm Visual Analogue Scale. The visual analogue scales range from 'would feel very anxious' to 'would feel very confident.' The point assigned on the visual analog scale is measured and scored from zero up to 100. The 23-item SEPC has three sub-scales of a) Communication (8 items; score ranging from 0 to 800); b) Patient management (8 items; score ranging from 0 to 800); c) Multi-disciplinary team working (7 items; score ranging from 0 to 700).

Thanatophobia Scale (11): The original TS has seven items and assesses attitudes related to the care for dying patients. The scale offers a reliable measure of students' outcome expectancy related to PC (6). Participants rate how much they agree/disagree with expressed negative attitudinal statements, with the final scores ranging from 7 to 49. Higher ratings indicate higher thanatophobia levels.

Both original scale was translated and validated to Brazilian Portuguese before this study (27).

For peer review only - http://bmjopen.bmj.com/site/about/guidelines.xhtml

Ethics

This research was conducted following the Declaration of Helsinki. We assured any student who was uncomfortable with the research enquiring would not feel obliged to participate. Themes related to death could be emotive and/or sensitive; thus, students could contact the researchers to receive support or referral if/as required. The Research Ethics Committee of both medical schools approved the study (58198016.4.0000.5404/2016; 58198016.4.3001.5411/2016).

Patient and Public Involvement

We did not involve patients directly in this study.

Data Analysis

Continuous variables were described by mean, minimal and maximal values, and categorical variables as absolute (n) and relative frequency (%). We analysed the difference between students' pre and post main scores on the scales using t-tests. Effect size was calculated by Cohen's d and used for checking the amplitude of changes in pre- and post-test scores, providing an index to assess the magnitude of the difference detected on t-test results. The effect size was characterized as small, medium, large, or very large and evaluated the magnitude of change despite the sample size (28). Bonferroni was used to correct the alpha value (29). Therefore, we used alpha value of 0.0125 for all analyses. Data were analysed using IBM-SPSS 21.0 (30).

Results

From the total students invited, two students from each school were excluded due to incomplete data. Thus, we included 78 students from MS1 and 56 students in MS2 (65.0% and 62.2% of last year students, respectively). Students' demographic data and

For peer review only - http://bmjopen.bmj.com/site/about/guidelines.xhtml

previous experiences are summarized in Table 1, which were answered in pre-test data collecting.

Table 1 - Descriptive data of MS1 and MS2

	Medical School 1	Medical School 2
**Sample size**	78	56
**Average age in years (min – max)**	24.8 (22-30)	25.2 (22-34)
**Gender**		
Male	36 (46.2%)	30 (53.6%)
Female	42 (53.8%)	26 (46.4%)
Has anybody that you have been close to died?		
Yes	63 (80.8%)	45 (80.4%)
No	15 (19.2%)	11 (19.6%)
Have you ever witnessed the moment of a person's death?		
Yes	64 (82.1%)	52 (92.9%)
No	14 (17.9%)	4 (7.1%)
During your professional training, have you ever taken care of anybody that eventually died?		
Yes	75 (96.2%)	55 (98.2%)
No	3 (3.8%)	1 (1.8%)

In both schools, students had a significant increase in self-efficacy in all dimensions of the SEPC ($p<0.001$ for all analyses). Students' thanatophobia levels significantly decreased in MS1, but not in MS2, as showed in Table 2 (significant results are highlighted). The effect size of the change in SEPC was larger at MS1 when compared to MS2. The effect size magnitude of TS was moderate in MS1.

For peer review only - http://bmjopen.bmj.com/site/about/guidelines.xhtml

Table 2 – SEPC and Thanatophobia score means, t-test and effect size at Medical School 1 and 2

Instruments	***Pre-test***	***Post-test***	***Difference Post - Pre***	***t***	***p***	***CI95%***	***Cohen's d***	***Effect Size Magnitude***
**Medical School 1 (n=78)**								
SEPC - Communication	346.50 (145.17)	573.90 (124.47)	227.39 (138.50)	14.50	**<0.001**	**258.62, 196.17**	**1.64**	***Very large***
SEPC - Patient Management	338.38 (112.56)	527.50 (113.55)	189.11 (140.30)	11.90	**<0.001**	**220.75, 157.48**	***1.35***	***Very large***
SEPC - Multidisciplinary Teamwork	361.26 (124.28)	470.35 (105.77)	109.09 (118.86)	8.11	**<0.001**	**135.17, 81.01**	***0.92***	***Large***
TS	21.54 (7.56)	16.54 (7.58)	-5.00 (5.91)	-7.06	**<0.001**	**3.59, 6.41**	***0.79***	***Moderate***
**Medical School 2 (n=56)**								
**SEPC - Communication**	296.41 (147.41)	398.00 (171.64)	101.59 (151.30)	5.02	**<0.001**	**142.11, 61.07**	**0.67**	***Moderate***
**SEPC - Patient Management**	327.86 (133.91)	462.07 (126.55)	134.21 (132.69)	7.57	**<0.001**	**169.75, 98.68**	**1.01**	***Large***
**SEPC - Multidisciplinary Teamwork**	388.50 (120.18)	454.96 (120.49)	66.46 (136.82)	3.63	**=0.001**	**103.10, 29.83**	**0.49**	***Small***
**TS**	22.82 (8.77)	21.20 (7.67)	-1.62 (5.45)	1.96	=0.056	-0.14, - 3.07	0.26	*Small*

Discussion

In this study, the SEPC and TS measured significant improvements in students' self-efficacy in palliative care and thanatophobia levels, after the placements analysed,

For peer review only - http://bmjopen.bmj.com/site/about/guidelines.xhtml

except in MS2, in which TS changes were not significant. These results support that non-specialized rotation can provide PC training with effects in students' self-efficacy in PC and thanatophobia. Most of the students in both schools informed some previous experience with dying people, or as a personal experience or during their professional training. This could suggest that previous clinical placements give opportunities for caring of dying patients. Nevertheless, the placements analysed in this study gave to students an additional opportunity to care of dying patients and purposely learn and discuss PC topics.

In both schools, the placements brought improvements in students` self-efficacy in palliative care, in all subscales, as expected after a learning experience that includes PC topics and discussions. Another study with UK medical students had similar results (8). Nevertheless, in the UK study, the effect size magnitude was larger in almost every subscale of SEPC, although the pre-test results were similar. One explanation for this could be the specialized PC placement the UK students had. There are other factors to be considered: time dedicated to PC training, specialized supervisors and services, placement length and the learning environment. In this case, a specialized placement, focused on training PC competencies, could bring development to students' self-efficacy and decrease their thanatophobia. In MS1, a decreasing in TS was observed, but not in MS2. Maybe this is a consequence of the experience in MS1 during the placement is related to more experience with critical and dying patients (see Appendix).

Worldwide, medical educators have devised different strategies for PC training, and there is no 'gold standard' to guide best practices (12,31,32). Although training in specialized PC services could bring larger improvement in SEPC, these services are not common in Brazil and are not an option for training medical students. Including a PC specialized placement in the already busy and crowded medical curriculum poses a challenge for medical educators. The challenge has two edges; one is the scarcity of specialized services and professionals to facilitate training, particularly in the developing

For peer review only - http://bmjopen.bmj.com/site/about/guidelines.xhtml

world (where the PC is still incipient and needs substantial development); the second is the lack of space in the curriculum.

Ultimately, clinicians in most specialties, such as internal medicine, geriatrics, oncology, emergency medicine, intensive care, primary care, paediatrics, and so on, will have PC patients among their caseloads. Although there can often be a lack of PC services, different disciplines and placements should include PC in their curricula and provide PC training for all medical students (33). Teachers could customize different clinical placements to modulate learning possibilities to students considering the resources available as showed in this study medical schools. By doing so, medical educators could ensure a psychological safety environment for learning, including feedback routines and the development of self-efficacy, which we considered as a fundamental aspect in schools we analysed (34). Our study showed that non-specialized rotations, which include specific topics in PC, were capable of improving students` self-efficacy in PC and decreasing their thanatophobia levels.

Therefore, others aspects as learning environment and experience, feedback opportunities and the inclusion of PC as part of clinical caring, could play relevant elements for PC training in this context (19). We believe that this finding is related to opportunities to reflect on real end-of-life cases that were provided for the students during these placements. As anticipated by Kolb`s experiential learning theory, the several occasions to reflect in and on the practice could have nurtured confidence and a readiness to practice PC (19). In fact, the social cognitive theory states that self-efficacy and outcome expectancy are both modulated by the feedback (perceived support) from supervisors, which will eventually, impact on the internalization of the desired behaviour (5). Possibly offering opportunities to experience and reflect, guided by qualified supervision with structured feedback, may be as important as having access to specialized PC centres for the learning of undergraduate students (5,7).

The challenge posed to medical educators is building up a meaningful learning process in complex workplaces in different clinical rotations, despite the lack of ideal

For peer review only - http://bmjopen.bmj.com/site/about/guidelines.xhtml

circumstances (18). Clinical teachers need to nurture the learning experience of students embracing the contextual elements, even the negative ones, as teaching assets and not as pitfalls. Students will practice medicine in the real world and need to learn how to deal with the context of the workplace. Those with higher self-efficacy are more likely to reflect on their challenges and experiences to improve performance (5). In this sense, reflection is essential not only to learn but also to feel the urge to change and improve the healthcare delivered to patients (9). Therefore, including structured PC discussions in clinical rotations may offer an opportunity for teachers not only to prepare students to provide proper palliative care but also to open the debate about the relevance of including PC into their future practice.

Strengths and limitations

This study had a consistent methodological approach and involved two different medical schools, with different undergraduate curricula, which included PC training in non-specialized rotations. We demonstrated that, although the two groups of students had different approaches in the sense of learning PC, both groups had improvements in their self-efficacy, even when students did not have access to specialized PC services.

Nevertheless, we should acknowledge some limitations in our study. Firstly, we did not access students' conceptual knowledge of PC, neither the effects of other factors as placement length nor learning approaches. In this sense, as we did not control other factors, we cannot conclude if the changes in scales are related to daily placements` clinical situations or to specific teaching approaches. An interventional study with control groups would be more appropriate to verify if and how the different educational approaches could have different impacts on self-efficacy in palliative care and thanatophobia levels.

Secondly, although the students have expressed more confidence in their performance, we cannot conclude that the improvements in SEPC and thanatophobia, observed in this

For peer review only - http://bmjopen.bmj.com/site/about/guidelines.xhtml

study, will be persistent for the actual medical practice of the students. The proper assessment for this will require further evaluation over the new doctors in their practice. Also, improvements in self-efficacy, in general, can be related to the opportunity given to medical students to reflect on their clinical experiences under the guidance of motivated facilitators (5). This general improvement could have also been extrapolated to Palliative Care competencies (7). Additionally, in MS2, as one of the researchers was supervisor, a socially desirable answer from the participants cannot be excluded.

Furthermore, the findings in this study are limited to two Brazilian medical schools and any generalization should be cautious. Future studies are needed to verify whether our findings will be consistent across different contexts.

Implications for practice and next steps

In a context of an overloaded curriculum and a lack of departments and services of palliative medicine in medical schools, our findings suggest that inserting PC topics in different non-specialized rotations could benefit the development of medical students self-efficacy in PC and reduce their thanatophobia. Thus, a vertical insertion of PC concepts and practices along the course, in different disciplines and rotations, could address PC learning needs for undergraduate students, even without specialized PC services for medical training.

We still need to understand the impact of implementing PC training on clinical rotations on students` declarative and procedural knowledge acquisition. It is also essential to clarify what are the teaching methodologies related to better development of PC competencies in undergraduate medical students.

Conclusion

Our findings support that non-specialized rotations can include PC basic competencies as part of the training. Medical schools could prepare future doctors to provide PC in any

For peer review only - http://bmjopen.bmj.com/site/about/guidelines.xhtml

area it is required by embracing palliative care as a core competence, indispensable for becoming a doctor.

Glossary Terms

Palliative care: "approach that improves the quality of life of patients and their families facing the problem associated with life-threating illness, through the prevention and relief of suffering by means of early identification and impeccable assessment and treatment of pain and other problems, physical, psychosocial and spiritual."(2)

Self-efficacy: "the confidence to carry out the courses of action necessary to accomplish desired goals" (35)**.**

Thanatophobia: death anxiety that caregivers and healthcare professionals may feel when caring for dying patients (11).

Declarations

Ethics approval and consent to participate

This research was conducted following the Declaration of Helsinki. We assured any student who was uncomfortable with the research enquiring would not feel obliged to participate. Themes related to death could be emotive and/or sensitive; thus, students could contact the researchers to receive support or referral if/as required. The Research Ethics Committee of both medical schools approved the study (58198016.4.0000.5404/2016; 58198016.4.3001.5411/2016).

Consent for publication

Not applicable

Availability of data and materials

The datasets used and analysed during the current study are available from the corresponding author on reasonable request.

For peer review only - http://bmjopen.bmj.com/site/about/guidelines.xhtml

Competing interests

GAMB is a supervisor in MS2 and participate in collecting data.

The other authors declare that they have no competing interests.

Funding

This work was supported by CAPES, grant number 88881.188776/2018-01 (PhD sandwich scholarship – Edictal 47/2017) to the first author for analysis and manuscript writing.

Author contributions

GG, SRM and MACF conceived and designed the study. GG and GAMB collected the data. GG, DCF and MACF analysed the data. GG and MACF were the major contributors in manuscript writing. DCF, GAMB and SRM provided meaningful inputs and critical review of the manuscript. All authors read and approved the final manuscript.

Acknowledgements

Palliative Care Institute Liverpool, the University of Liverpool for hosting and supporting the manuscript writing;

Medical students and supervisors involved on data collection;

Author's information

GG, M.Sc., M.D., G.P. is a PhD student at the School of Medical Sciences, UNICAMP, Brazil, in the field of medical education focused on palliative care education;

DCF, PhD is a young investigator at Department of Medical Psychology and Psychiatry, School of Medical Sciences, UNICAMP, Brazil;

GAMB, MD, MSc, Ph.D. is Associate Professor, Anaesthesiology Department, Botucatu Medical School, Sao Paulo State University - UNESP, Botucatu-SP, Brazil

For peer review only - http://bmjopen.bmj.com/site/about/guidelines.xhtml

SRM, PhD is Research Lead at Palliative Care Institute Liverpool, University of Liverpool, Liverpool, UK;

MACF, M.D., PhD is Associate Professor, Life and Health Sciences Research Institute (ICVS), Medical School, University of Minho, Portugal and Senior Researcher at Center for Education Development and Research in Health Professions (CEDAR), University of Groningen, Groningen, the Netherlands;

References

1. Marinho F, de Azeredo Passos VM, Carvalho Malta D, Barboza França E, Abreu DMX, Araújo VEM, et al. Burden of disease in Brazil, 1990–2016: a systematic subnational analysis for the Global Burden of Disease Study 2016. Lancet [Internet]. 2018 [cited 2018 Dec 14];392(10149):760–75. Available from: https://vizhub.

2. Connor SR, Sepulveda Bermedo MC. Global atlas of palliative care at the end of life [Internet]. World Palliative Care Alliance. 2014. 111 p. Available from: http://www.who.int/nmh/Global_Atlas_of_Palliative_Care.pdf

3. Murtagh FE, Bausewein C, Verne J, Groeneveld EI, Kaloki YE, Higginson IJ. How many people need palliative care? A study developing and comparing methods for population-based estimates. Palliat Med [Internet]. 2014 [cited 2019 Mar 6];28(1):49–58. Available from: https://journals.sagepub.com/doi/pdf/10.1177/0269216313489367

4. Etkind SN, Bone AE, Gomes B, Lovell N, Evans CJ, Higginson IJ, et al. How many people will need palliative care in 2040? Past trends, future projections and implications for services. BMC Med [Internet]. 2017 [cited 2019 Mar 6];15:102. Available from: https://www.ncbi.nlm.nih.gov/pmc/articles/PMC5436458/pdf/12916_2017_Article_860.pdf

5. Artino AR. Academic self-efficacy: from educational theory to instructional practice. Perspect Med Educ [Internet]. 2012 [cited 2019 Feb 20];1(2):76–85.

For peer review only - http://bmjopen.bmj.com/site/about/guidelines.xhtml

Available from: https://link.springer.com/content/pdf/10.1007%2Fs40037-012-0012-5.pdf

6. Mason S, Ellershaw J. Assessing undergraduate palliative care education: Validity and reliability of two scales examining perceived efficacy and outcome expectancies in palliative care. Med Educ. 2004;38(10):1103–10.

7. Mason SR, Ellershaw J. Preparing for palliative medicine; evaluation of an education programme for fourth year medical undergraduates. Palliat Med [Internet]. 2008 [cited 2019 Feb 11];22:687–92. Available from: https://journals.sagepub.com/doi/pdf/10.1177/0269216308090070

8. Gilissen J, Pivodic L, Wendrich-van Dael A, Cools W, Stichele R Vander, Van den Block L, et al. Nurses' self-efficacy, rather than their knowledge, is associated with their engagement in advence care planning in nurisng homes: a syrvey study. Palliat Med. 2020;34(7):917–34.

9. Mason SR, Ellershaw J. Undergraduate training in palliative medicine: is more necessarily better? Palliat Med [Internet]. 2010 [cited 2019 Jan 29];24(3):306–9. Available from: https://journals.sagepub.com/doi/pdf/10.1177/0269216309351867

10. Neil H, Frey R, Gott M. Instruments used to measure the effectiveness of palliative care education initiatives at the undergraduate level: A systematic literature review. BMJ Support Palliat Care [Internet]. 2013 [cited 2019 Feb 11];3:114–9. Available from: http://www.sherpa.ac.uk/romeo/issn/2045-435X/https://researchspace.auckland.ac.nz/docs/uoa-docs/rights.htm

11. Merrill J, Lorimor R, Thornby J, Woods A. Caring for Terminally ILL Persons: Comparative Analysis of Attitudes (Thanatophobia) of Practicing Physicians, Student Nurses, and Medical Students. Psychol Rep. 1998;

12. Fitzpatrick D, Heah R, Patten S, Ward H. Palliative Care in Undergraduate Medical Education-How Far Have We Come? Am J Hosp Palliat Med [Internet]. 2017 [cited 2019 Mar 22];34(8):762–73. Available from:

For peer review only - http://bmjopen.bmj.com/site/about/guidelines.xhtml

https://journals.sagepub.com/doi/pdf/10.1177/1049909116659737

13. Padilha De Toledo A, Gonçalves Priolli D. Cuidados no Fim da Vida: O Ensino Médico no Brasil (End-of-life Care Education in Brazilian Medical Schools). Rev Bras Educ Med [Internet]. 2012 [cited 2019 Feb 28];36(1):109–17. Available from: http://www.scielo.br/pdf/rbem/v36n1/a15v36n1.pdf

14. Carrasco JM, Lynch TJ, Garralda E, Woitha K, Elsner F, Filbet M, et al. Palliative Care Medical Education in European Universities: A Descriptive Study and Numerical Scoring System Proposal for Assessing Educational Development. J Pain Symptom Manage [Internet]. 2015 Oct 1 [cited 2019 Feb 28];50(4):516-523.e2. Available from: https://www.sciencedirect.com/science/article/pii/S0885392415002419

15. Vindrola-Padros C, Mertnoff R, Lasmarias C, Gómez-Batiste X. Palliative care education in Latin America: A systematic review of training programs for healthcare professionals. Palliat Support Care. 2018;16(1).

16. Análise Situacional e Recomendações para Estruturação de Programas de Cuidados Paliativos no Brasil. São Paulo: Academia Nacional de Cuidados Paliativos; 2018.

17. Oliveira JR de, Ferreira AC, Rezende NA de. Ensino de bioética e cuidados paliativos nas escolas médicas do Brasil. Rev Bras Educ Med [Internet]. 2013 [cited 2018 Dec 12];37(2):285–90. Available from: http://www.scielo.br/pdf/rbem/v37n2/17.pdf

18. Yardley S, Teunissen PW, Dornan T. Experiential learning: AMEE Guide No. 63. Med Teach. 2012 Feb;34(2).

19. Taylor DCM, Hamdy H. Adult learning theories: Implications for learning and teaching in medical education: AMEE Guide No. 83. Med Teach. 2013 Nov;35(11).

20. Medicina - Ranking de Cursos - Ranking Universitário Folha - 2018 [Internet]. [cited 2019 May 9]. Available from: https://ruf.folha.uol.com.br/2018/ranking-de-

For peer review only - http://bmjopen.bmj.com/site/about/guidelines.xhtml

cursos/medicina/

21. Universities | Top Universities [Internet]. [cited 2019 May 9]. Available from: https://www.topuniversities.com/universities/country/brazil/subject/medicine

22. Schweller M, Ribeiro DL, Passeri SR, Wanderley JS, Carbalho-Filho MA. Simulated medical consultations with standardized patients: In-depth debriefing based on dealing with emotions. Rev Bras Educ Med [Internet]. 2018 [cited 2019 Jun 13];42(1):82–91. Available from: http://dx.doi.org/10.1590/1981-52712018v42n1RB20160089

23. Schweller M, Costa FO, Antonio MAR, Amaral EM, Carvalho-Filho MA de. The impact of simulated medical consultations in the empathy levels of students at one medical school. Acad Med [Internet]. 2014 [cited 2019 Mar 5];89(4):632–7. Available from: https://www.ncbi.nlm.nih.gov/pmc/articles/PMC4885552/pdf/acm-89-632.pdf

24. Von Elm E, Altman DG, Egger M, Pocock SJ, Gøtzsche PC, Vandenbroucke JP. The Strengthening the Reporting of Observational Studies in Epidemiology (STROBE) Statement: Guidelines for reporting observational studies. BMJ. 2007;335:805–8.

25. Whyte R, Quince T, Benson J, Wood D, Barclay S. Medical students' experience of personal loss: incidence and implications. BMC Med Educ [Internet]. 2013 [cited 2019 Mar 4];13:36. Available from: http://www.biomedcentral.com/1472-6920/13/36

26. Barclay S, Whyte R, Thiemann P, Benson J, Wood DF, Parker RA, et al. An important but stressful part of their future work: Medical students' attitudes to palliative care throughout their course. J Pain Symptom Manage [Internet]. 2015;49(2):231–42. Available from: http://dx.doi.org/10.1016/j.jpainsymman.2014.06.004

27. Gryschek G, Cecilio-Fernandes D, Mason S, de Carvalho-Filho MA. Assessing palliative care education in undergraduate medical students: translation and

For peer review only

validation of the Self-Efficacy in Palliative Care and Thanatophobia Scales for Brazilian Portuguese. BMJ Open. 2020;10(6):e034567.

28. Sullivan GM, Feinn R. Using Effect Size-or Why the P Value Is Not Enough. [cited 2019 Apr 8]; Available from: http://dx.doi.org/10.4300/JGME-D-12-00156.1

29. Bland JM, Altman DG. Multiple significance tests: the Bonferroni method. BMJ. 1995;310(170).

30. IBM Corp. IBM SPSS Statistics for Windows. Armonk, NY: IBM Corp.; 2012.

31. Field D, Wee B. Preparation for palliative care: Teaching about death, dying and bereavement in UK medical schools 2000-2001. Med Educ. 2002;36(6):561–7.

32. Schulz-Quach C, Wenzel-Meyburg U, Fetz K. Can e-learning be used to teach palliative care?-medical students' acceptance, knowledge, and self-estimation of competence in palliative care after elearning. BMC Med Educ [Internet]. 2018 [cited 2019 Mar 22];18(82). Available from: https://doi.org/10.1186/s12909-018-1186-2

33. Lloyd-Williams M, Macleod RDM, Macleod RD. A systematic review of teaching and learning in palliative care within the medical undergraduate curriculum A systematic review of teaching and learning in palliative care within the medical undergraduate curriculum. Med Teach [Internet]. 2004 [cited 2019 Feb 12];26(8):683–90. Available from: https://doi.org/10.1080/01421590400019575

34. Caverzagie KJ, Goldenberg MG, Hall JM. Psychology and learning: The role of the clinical learning environment. Med Teach [Internet]. 2019;41(4):375–9. Available from: https://doi.org/10.1080/0142159X.2019.1567910

35. Klassen RM, Klassen JRL. Self-efficacy beliefs of medical students: a critical review. Perspect Med Educ [Internet]. 2018 [cited 2019 Feb 20];7(2):76–82. Available from: https://doi.org/10.1007/s40037-018-0411-3

For peer review only - http://bmjopen.bmj.com/site/about/guidelines.xhtml

Appendix

Survey model/ Portuguese-BR version of SEPC and Thanatophobia Scale	
Original Questions	*English Translation*
Você já perdeu na morte alguém que, segundo a sua percepção, teve um papel importante na sua vida?	**Has anybody that you have been close to died?**
Você já viu alguém morrendo?	**Have you ever witnessed a person's death?**
Durante sua formação profissional, você já participou do cuidado de alguém que acabou morrendo?	**During your professional training, have you ever taken care of anybody that eventually died?**

For peer review only

Medical Schools` Placements

In **Medical School 1**, students rotate in three different clinical environments. In the first 4 weeks, they stay in the Emergency Department. For an international audience, it is important to explain that Emergency Departments in Brazil are chaotic environments that are always working 4-5 times above their capacities. For instance, in medical school 1, the Emergency Department was planned to have 25 patients in observation but end up admitting more than 100. Ideally, patients should stay there for 24 hours maximum, but occasionally they stay there for up to two weeks. Even patient who need intensive care can stay in the Emergency Department for up to 5 days waiting for a vacancy inside the hospital. Students engage actively in the process of care and take direct responsibility for patients, always under the supervision of an experienced physician. In the following 4 weeks, students stay for 2 weeks in an ICU, and 2 weeks in an internal medicine with a high level of complexity, both devoted to patients admitted through the Emergency Department. In both, the internal medicine ward and ICU, in the morning, students are responsible for presenting the case of the patients during the daily clinical round and for organizing and implementing the therapeutic plan that is discussed together with the multi-professional team. On Mondays and Fridays mornings, after the round, students engage in a clinical ethics discussion about one of the patients they are caring for. In the afternoon, on Wednesdays and Thursdays, students engage in simulation activities dedicated to address communication issues and develop emotional competence. Every Friday afternoon, students have discussions about death and dying and palliative care. The structure of the weeks in the mentioned rotations are shown below.

**MS1 – Week Planning**	**Monday**	**Tuesday**	**Wednesday**	**Thursday**	**Friday**
**Weeks 1 to 4 Morning**	Emergency Department	Emergency Department	Emergency Department	Emergency Department	Emergency Department
**Afternoon**	Emergency Department	Emergency Department	Emergency Department	Emergency Department	Emergency Department
**Weeks 4 to 8 Morning**	ICU Clinical Round Ethics Discussion	ICU Clinical Round	ICU Clinical Round	ICU Clinical Round Ethics Discussion	ICU Clinical Round
**Afternoon**	Lectures	Lectures	Simulation Sessions	Simulation Sessions	Discussions about death and dying

Medical School 1 – Placement Summary

**Placement**	Emergency and Critical Care Medicine
**Length**	8 weeks
**Program hours**	440 hours
**Learning Outcomes related to Palliative Care**	- Identify terminal patients and understand the moment to shift from a predominant curative approach to a predominant palliative approach.<br>- Provide end-of-life care to terminal patients<br>- Understand the concepts of ortothanasia and dysthanasia<br>- Understand and apply the ethical principles of surrogate decision-making<br>- Understand and apply the principles of interprofessional collaboration in the context of end-of-life care<br>- Understand how the different cultural perspectives related to death and dying impact on end-of-life care<br>- Understand how our own perspectives on death and dying may interfere with the end-of-life care that we provide to our patients<br>- Develop patient-centered communication based on presence, improvisation, and empathy.
**Groups size**	14 students
**Learning activities**	- Daily rounds to: emergency department and critical care wards;<br>- Case discussion sessions;<br>- High fidelity simulation sessions: emergency room and consultation scenarios;<br>- Point-of-care ultrasound classes and bedside training;<br>- Remote learning: digital educational plataform Moodle;<br>- Mechanical ventilation classes;<br>- Palliative care classes;<br>- Palliative care ethics discussions;
**Palliative care topics**	- Breaking bad news;<br>- End-of-life ethics;<br>- Thanatology;
**Total Supervisors**	All Medical doctors<br>- Emergency department: 8<br>- Internal Medicine Ward: 2<br>- Intensive Care Unit: 2
**Supervisors experience in Medical Education**	The course coordinator is a researcher in medical education, and 2 of the eight supervisors have a Ph.D. in medical education focused on developing new active learning strategies. All the supervisors have consistently participated in medical education congresses, presenting both research and educational innovations.
**Supervisors experience in Palliative Care**	Lecturer in Palliative care topics<br>End of life care ethics discussions in weekly basis;

For peer review

**Educational Methods**	- Daily wards rounds;<br>- Blended methods: classes and remote learning;<br>- Bedside teaching;<br>- Simulation sessions;<br>- Clinical case discussions;
**Learning activities related to PC and end-of-life care**	- Clinical simulation targeting breaking bad news, communication skills, and emotional competence (4 sessions of 4h each);<br>- End of Life Ethics discussions (4 sessions of 1 hour each);
**Teaching highlights**	- Simulation and feedback<br>- Reflexive learning<br>- Case discussion of complex and critical patients
**Multidisciplinary team coworking**	Coworking close to other medical specialties: psychiatry, neurology, orthopedics and traumatology
**Supervisors Feedback routine**	Daily feedback in wards rounds and other learning activities, such as Moodle and simulation sessions.<br>Grades mailing in the end of the placement with all evaluation results
**Evaluation**	Multidimensional evaluation<br>- Two theorical classic tests: multiple choice and open questions<br>- OSCE: clinical scenarios based in placement daily situations<br>- Supervisors, peers and self-evaluation on attitudes and behavior

In **Medical School 2** the Anesthesiology and Pain Medicine placement have a total of 280 hours of activities. About 140 hours of this workload is related to palliative care topics along pain medicine learning activities. The group of students are divided into sub-groups composed of 2 students to palliative care activities, which are performed during home visits, in wards and in outpatient clinics, assisting patients under palliative care. All these clinical-practical activities take place in multiprofessional settings with the presence of nurses, social workers and other professionals. At the same time there are theoretical activities in which subjects such as the treatment of acute and chronic pain, bases of palliative care and health communication are addressed, with an emphasis on bad news.

**MS2 – Week Planning**	**Monday**	**Tuesday**	**Wednesday**	**Thursday**	**Friday**
**Weeks 1 to 3 Morning**	Anesthesiology department	Anesthesiology department	Anesthesiology department	Anesthesiology department	Anesthesiology department
**Afternoon**	Anesthesiology department	Anesthesiology department	Anesthesiology department	Anesthesiology department	Anesthesiology department
**Weeks 3 to 6 Morning**	Pain and Palliative Care Outpatient Clinic	Pain and Palliative Care Outpatient Clinic	Pain and Palliative Care Outpatient Clinic	Pain and Palliative Care Outpatient Clinic	Pain and Palliative Care Outpatient Clinic
**Afternoon**	Lecture	Lecture	Clinical case discussion	Pain and Palliative Care Outpatient Clinic	Homecare

For peer review only

Medical School 2 – Placement Summary

**Clinical placement**	Anesthesiology and Pain Medicine
**Length**	6 weeks
**Total program hours**	280 hours
**Learning Outcomes related to Palliative Care**	- Identify patients who can benefit from palliative care<br>- Understand the concepts of palliative care<br>- Understand the importance of adequate communication in health and symptoms management<br>- Evaluate and manage acute and chronic pain
**Groups size**	12 students
**Learning activities**	- Traditional and interactive lectures using Kahoot (game-based learning platform)<br>- Patient care in outpatient clinic, in the hospital yards and home care<br>- Clinical cases discussion
**Palliative care topics**	- Palliative care concepts<br>- Communication in palliative care<br>- Pain and symptom management
**Total Supervisors**	- Medical doctors: 2<br>- Registered Nurses: 2
**Supervisors experience in Medical Education**	Medical Doctors are PhD, working as clinical teachers at the medical school for more than 10 years
**Supervisors experience in Palliative Care**	Medical Doctors are board-certificated palliative care specialists<br>Registered Nurses have no specific training
**Educational Methods**	- Lectures<br>- Clinical case discussions<br>- Training procedures skills<br>- Clinical exposure to palliative patient care
**Learning activities related to PC and end-of-life care**	- PC lectures (8h): concepts, communications skills, symptoms management<br>- Clinical discussions of outpatient patients (20h)<br>- Homecare visits (4h)
**Teaching highlights**	- Lectures<br>- Case discussions of chronic patients<br>- Working in a multi-professional healthcare team specialized in Palliative Care
**Multidisciplinary team coworking**	Discussion of clinical home care cases with the participation of registered nurse, social worker, psychologist and nutritionist. Physical therapists are available under referral
**Supervisors Feedback routine**	During daily practical activities
**Evaluation**	Classical style tests and clinical skill practical evaluation using simulated setting activities

For peer review only

Reporting checklist for cohort study.

Based on the STROBE cohort guidelines.

Instructions to authors

Complete this checklist by entering the page numbers from your manuscript where readers will find each of the items listed below.

Your article may not currently address all the items on the checklist. Please modify your text to include the missing information. If you are certain that an item does not apply, please write "n/a" and provide a short explanation.

Upload your completed checklist as an extra file when you submit to a journal.

In your methods section, say that you used the STROBE cohortreporting guidelines, and cite them as:

von Elm E, Altman DG, Egger M, Pocock SJ, Gotzsche PC, Vandenbroucke JP. The Strengthening the Reporting of Observational Studies in Epidemiology (STROBE) Statement: guidelines for reporting observational studies.

		Reporting Item	Page Number
**Title and abstract**			
Title	#1a	Indicate the study’s design with a commonly used term in the title or the abstract	1
Abstract	#1b	Provide in the abstract an informative and balanced summary of what was done and what was found	2
**Introduction**			
Background / rationale	#2	Explain the scientific background and rationale for the investigation being reported	3-5
Objectives	#3	State specific objectives, including any prespecified hypotheses	6
**Methods**			

For peer review only

Study design	#4	Present key elements of study design early in the paper	7
Setting	#5	Describe the setting, locations, and relevant dates, including periods of recruitment, exposure, follow-up, and data collection	7-10
Eligibility criteria	#6a	Give the eligibility criteria, and the sources and methods of selection of participants. Describe methods of follow-up.	6-8
Eligibility criteria	#6b	For matched studies, give matching criteria and number of exposed and unexposed	n/a
Variables	#7	Clearly define all outcomes, exposures, predictors, potential confounders, and effect modifiers. Give diagnostic criteria, if applicable	9
Data sources / measurement	#8	For each variable of interest give sources of data and details of methods of assessment (measurement). Describe comparability of assessment methods if there is more than one group. Give information separately for for exposed and unexposed groups if applicable.	9
Bias	#9	Describe any efforts to address potential sources of bias	8, 9
Study size	#10	Explain how the study size was arrived at	8
Quantitative variables	#11	Explain how quantitative variables were handled in the analyses. If applicable, describe which groupings were chosen, and why	9
Statistical methods	#12a	Describe all statistical methods, including those used to control for confounding	10
Statistical methods	#12b	Describe any methods used to examine subgroups and interactions	10
Statistical methods	#12c	Explain how missing data were addressed	10
Statistical methods	#12d	If applicable, explain how loss to follow-up was addressed	10
Statistical methods	#12e	Describe any sensitivity analyses	n/a

Results

For peer review only

Participants	#13a	Report numbers of individuals at each stage of study—eg numbers potentially eligible, examined for eligibility, confirmed eligible, included in the study, completing follow-up, and analysed. Give information separately for for exposed and unexposed groups if applicable.	10
Participants	#13b	Give reasons for non-participation at each stage	10
Participants	#13c	Consider use of a flow diagram	n/a
Descriptive data	#14a	Give characteristics of study participants (eg demographic, clinical, social) and information on exposures and potential confounders. Give information separately for exposed and unexposed groups if applicable.	10, 11
Descriptive data	#14b	Indicate number of participants with missing data for each variable of interest	10
Descriptive data	#14c	Summarise follow-up time (eg, average and total amount)	n/a
Outcome data	#15	Report numbers of outcome events or summary measures over time. Give information separately for exposed and unexposed groups if applicable.	11-12
Main results	#16a	Give unadjusted estimates and, if applicable, confounder-adjusted estimates and their precision (eg, 95% confidence interval). Make clear which confounders were adjusted for and why they were included	11-12
Main results	#16b	Report category boundaries when continuous variables were categorized	11-12
Main results	#16c	If relevant, consider translating estimates of relative risk into absolute risk for a meaningful time period	n/a
Other analyses	#17	Report other analyses done—e.g., analyses of subgroups and interactions, and sensitivity analyses	n/a
**Discussion**			
Key results	#18	Summarise key results with reference to study objectives	12,13
Limitations	#19	Discuss limitations of the study, taking into account sources of potential bias or imprecision. Discuss both direction and magnitude of any potential bias.	15

For peer review only

Interpretation	#20	Give a cautious overall interpretation considering objectives, limitations, multiplicity of analyses, results from similar studies, and other relevant evidence.	13-15
Generalisability	#21	Discuss the generalisability (external validity) of the study results	16
**Other Information**			
Funding	#22	Give the source of funding and the role of the funders for the present study and, if applicable, for the original study on which the present article is based	18

The STROBE checklist is distributed under the terms of the Creative Commons Attribution License CC-BY. This checklist was completed on 02 August 2020 using https://www.goodreports.org/, a tool made by the EQUATOR Network in collaboration with Penelope.ai

For peer review only
